# Targeting ANGPTL3 and IL‐33/ST2 Ameliorates Diabetic Kidney Disease by Reducing Lipotoxicity, Alleviating Inflammation and Inhibiting Fibrosis

**DOI:** 10.1002/advs.75756

**Published:** 2026-05-19

**Authors:** Zhuojin Li, Zhonglian Cao, Rongrui Zhou, Xu Cheng, Xiaozhi Hu, Shuwen Xu, Zihan Dou, Ling Du, Dianwen Ju

**Affiliations:** ^1^ School of Pharmaceutical Sciences and Shanghai Engineering Research Center of Immunotherapeutics Qidong‐Fudan Innovative Institute of Medical Sciences Fudan University Shanghai China; ^2^ Minhang Hospital Fudan University Shanghai China; ^3^ Shanghai Geriatrics Medical Center Minhang Branch of Zhongshan Hospital Fudan University Shanghai China

**Keywords:** angiopoietin‐like protein 3, diabetic kidney disease, fusion protein, group 2 innate lymphoid cells, inflammatory response, interleukin‐33, metabolism disorder, sST2

## Abstract

Current therapies for diabetic kidney disease (DKD) targeting hyperglycemia and hypertension fail to halt progression, urging exploration of additional drivers. Renal lipotoxicity and chronic inflammation form a self‐perpetuating cycle driving DKD, yet multitarget interventions remain underexplored. We hypothesized that simultaneous inhibition of ANGPTL3 and IL‐33, two key regulators of lipid metabolism and the inflammatory‐fibrotic axis, would ameliorate DKD, and that integrating both antagonistic activities into a single bifunctional molecule offers translational advantages. We engineered a bifunctional fusion protein, FD03‐sST2, comprising an anti‐ANGPTL3 nanobody fused to the IL‐33 decoy receptor sST2. In high‐fat diet‐fed db/db mice, FD03‐sST2 significantly improved renal function (ACR, BUN, urine volume), reduced serum/hepatic lipids, and attenuated renal lipid accumulation. Mechanistically, it suppressed renal inflammation via NF‐κB/NLRP3 inhibition and ameliorated fibrosis by suppressing the IL‐33/ST2/ILC2 axis (reducing renal IL‐33/GATA3 signals and inhibiting IL‐33‐induced profibrotic factors from ILC2s). Transcriptomic and metabolomic analyses confirmed attenuation of DKD‐associated dysregulation. This study identifies a DKD cascade wherein lipotoxicity triggers IL‐33 release, amplifying injury through inflammation and fibrosis. By targeting ANGPTL3 and IL‐33 simultaneously, FD03‐sST2 interrupts this vicious cycle at two nodes—improving lipid metabolism while suppressing downstream inflammatory and fibrotic signaling—providing an integrated alternative to separate biologics.

## Graphical abstract

Dual targeting of ANGPTL3 and IL‐33/ST2 attenuates diabetic kidney disease by reprogramming lipid–inflammatory crosstalk. This strategy reduces renal lipotoxicity, suppresses inflammatory activation, and limits fibrotic remodeling, thereby preserving kidney structure and function and highlighting a mechanism‐guided therapeutic approach for metabolic kidney injury.

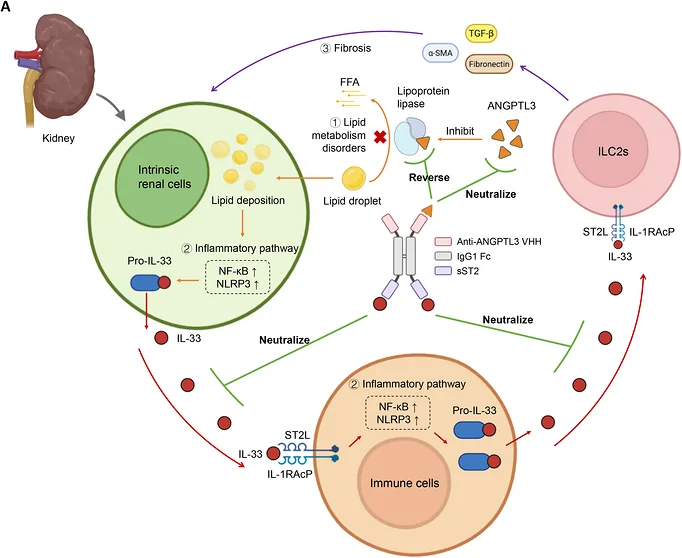

## 1 Introduction

Diabetic kidney disease (DKD) constitutes a leading contributor to end‐stage renal disease (ESRD) worldwide [1, 2], characterized by a complex pathogenic network in which metabolic dysfunction, chronic inflammation, and fibrosis form a vicious, self‐perpetuating loop [3, 4, 5]. This maladaptive cycle ultimately culminates in renal fibrosis, resulting in ESRD [1]. Despite therapeutic advances with agents including SGLT2 inhibitors and GLP‐1 receptor agonists, substantial clinical gaps remain in targeting the complex pathogenic mechanisms and progressive renal dysfunction associated with DKD [6, 7]. Consequently, investigating pathogenic networks and developing innovative therapies that can simultaneously target these fundamental pathological axes is essential for improving clinical outcomes.

Renal lipid accumulation plays a key role in the advancement of DKD, with resultant lipotoxicity being a primary emphasis in contemporary pathogenesis studies [8]. Hypertriglyceridemia and diminished high‐density lipoprotein cholesterol levels constitute well‐recognized independent predictors of progression to advanced chronic kidney disease (CKD) [9]. Therefore, rectifying lipid accumulation and glucose‐lipid metabolic abnormalities is crucial for decelerating the advancement of DKD [8]. Angiopoietin‐like protein 3 (ANGPTL3), a member of the angiopoietin‐like protein family [10, 11], contains a coiled‐coil domain (CCD) through which it governs lipid homeostasis via suppression of lipoprotein lipase (LPL) and endothelial lipase (EL) activity [12, 13, 14]. Serum ANGPTL3 levels are markedly increased in individuals with DKD, exhibiting a positive connection with ACR and negative correlation with GFR [15, 16]. In our previous research, we created a proprietary VHH‐Fc fusion protein that demonstrates a strong affinity for ANGPTL3 and remarkable stability [17, 18]. In a murine non‐alcoholic fatty liver disease (NAFLD) model, this agent markedly reduced circulating triglycerides (TG), total cholesterol (TC) and low‐density lipoprotein cholesterol (LDL‐C). Moreover, it decreased hepatic lipid buildup and liver damage in NAFLD murine models, thereby alleviating disease development. We propose that inhibiting ANGPTL3 activity may counteract ANGPTL3‐mediated suppression of LPL activity, thus alleviating the impact of chronic metabolic disorders on DKD.

Renal fibrosis is a critical element in the advancement of DKD to ESRD [1]. Postponing or obstructing the advancement of renal fibrosis can maintain renal function for an extended duration in individuals with DKD, thereby preventing its progression to ESRD [2, 19]. Group 2 Innate Lymphoid Cells (ILC2) are innate immune cells located in organs that closely resemble Th2 cells in both function and classification [20, 21]. These cells secrete effector cytokines (e.g., IL‐4, IL‐5, IL‐13) that orchestrate local immune and inflammatory reactions [20]. Recent research reveals that ILC2 cells are significant effector cells contributing to tissue fibrosis [22]. In a murine model of bleomycin‐induced pulmonary fibrosis, ILC2s upregulate and secrete TGF‐β, a key profibrotic mediator in lung tissue [23, 24]. These findings suggest that ILC2s are not merely associated with fibrosis, but can function as upstream profibrotic effector cells by producing mediators such as TGF‐β and by directly promoting matrix‐remodeling responses in stromal cells.

Interleukin‐33 (IL‐33), an IL‐1 family cytokine, is secreted by endothelial cells, epithelial cells, and fibroblasts under conditions of cellular stress, necrosis, or mechanical damage [25]. IL‐33 functions as “alarm factor” within the immune system, playing a pivotal role in inflammatory, infectious and autoimmune illnesses [26]. Suppression of tumorigenicity 2 (ST2, also known as *Il1rl1*) protein is the selective receptor for IL‐33, existing in a soluble secreted form (sST2) and a complete transmembrane form (ST2L) [27]. As an endogenous soluble decoy receptor for IL‐33, sST2 negatively modulates IL‐33‐related signaling pathways by competitively binding to IL‐33 [28], thereby preventing its engagement with membrane‐bound ST2L. The sST2‐based neutralization strategy offers several potential advantages over a direct anti‐IL‐33 neutralizing antibody approach: sST2 mimics a physiological regulatory mechanism, retains the flexibility to modulate rather than completely abrogate IL‐33 signaling, and carries a theoretically lower immunogenic risk as a non‐antibody endogenous protein scaffold [27]. ST2L is specifically expressed on the cell membrane of numerous immune cells participating in Type 2 immune responses [27]. Upon binding to IL‐33, it modulates Th2 cell‐mediated immune responses, mast cells and localized inflammatory reactions, exerting pro‐inflammatory effects and facilitating tissue repair responses [29, 30]. In a murine model of pulmonary fibrosis, an ILC2 subset expressing high levels of the IL‐33 receptor (*Il1rl1*) expanded within fibrotic lesions alongside IL‐33^+^ fibroblasts; conversely, mice deficient in ILCs or IL‐33 remained fibrosis‐free. In vitro cell co‐culture analyses demonstrated that activated ILC2s directly prompted fibroblasts to synthesize collagen following IL‐33 stimulation [31]. IL‐33 serves as a significant activator of ILC2 cells and is intricately associated with other inflammation‐related pathways [32, 33, 34]. It was demonstrated that IL‐33 enhances the transcriptional production of NLRP3 inflammasome constituents and its principal substrate pro‐IL‐1β, delivering the crucial “initial signal” for NLRP3 inflammasome activation [35, 36, 37]. We therefore hypothesized that IL‐33 contributes to DKD through two interconnected mechanisms: it amplifies NF‐κB/NLRP3‐dependent renal inflammation and activates ST2L‐expressing ILC2s, thereby driving a profibrotic program characterized by TGF‐β and extracellular‐matrix‐related factor production. Consequently, targeting IL‐33 treatment is anticipated to mitigate glomerular fibrosis and diminish inflammation.

We propose that DKD is propelled by a self‐sustaining pathogenic cycle that encompasses metabolic failure, chronic inflammation, and fibrosis. Integral to this process is renal lipid buildup and systemic dyslipidemia, which provoke lipotoxic damage and initiate the production of the alarmin IL‐33. This triggers a maladaptive inflammatory cascade marked by NF‐κB pathway activation and NLRP3 inflammasome assembly, which exacerbates renal inflammation and IL‐33 production. Excessive IL‐33 activates ILC2s, enhancing the release of pro‐fibrotic proteins that directly contribute to driving progressive renal fibrosis, the final common pathway to organ failure. We anticipated that concurrent targeting of ANGPTL3 and IL‐33 would improve DKD by correcting lipid metabolism and interrupting the downstream inflammatory‐fibrotic cascade, and that a bifunctional fusion format might be advantageous because it enforces fixed‐stoichiometry co‐delivery and simultaneous target engagement within a single therapeutic entity. To test this hypothesis, we engineered a novel bifunctional fusion protein, FD03‐sST2, comprising an anti‐ANGPTL3 nanobody (FD03) fused to the soluble IL‐33 decoy receptor sST2, thereby integrating lipid‐lowering and IL‐33‐neutralizing activities into one molecule and allowing direct comparison with the corresponding free‐drug combination. We then characterized its physicochemical properties and evaluated its biological activities. Subsequently, the in vivo therapeutic potential of FD03‐sST2 was assessed in a high‐fat diet‐induced DKD mouse model using db/db mice. The study examined not only glucose‐lipid metabolic parameters and NF‐κB/NLRP3 signaling, but also whether IL‐33‐driven activation of ILC2s constitutes a functional profibrotic node that can be interrupted by FD03‐sST2. Taken together, these results indicate that simultaneous targeting of ANGPTL3 and IL‐33 represents a promising therapeutic strategy for DKD.

## 2 Results

### 2.1 The Significant Up‐Regulation of ANGPTL3 and IL‐33 Expression in DKD Mice and Patients Suggested that they were Involved in the Progress of DKD Patients

**FIGURE 1:**
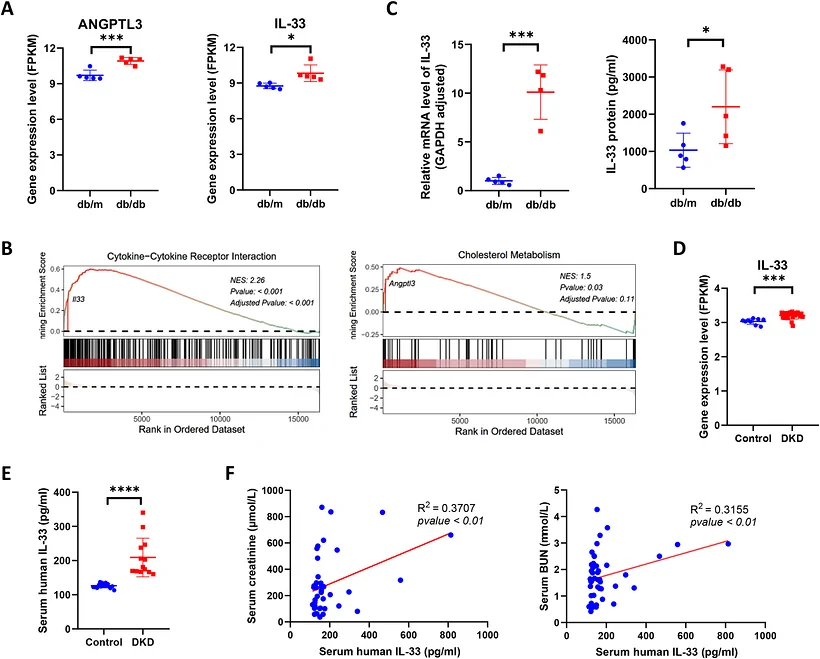
Expression of ANGPTL3 and IL‐33 in murine DKD models and patients with DKD. (A) Gene expression of ANGPTL3 and IL‐33 in control mice (n = 5) and DKD mice (n = 5). (B) GSEA of pathways related to negative regulation of ANGPTL3 and IL‐33. (C) *Il33* mRNA expression in kidney tissue and serum IL‐33 levels in control mice (n = 8) and DKD mice (n = 8). (D) IL‐33 gene expression in human controls (n = 9) and patients with DKD (n = 21). (E) Serum IL‐33 levels in human controls (n = 20) and patients with DKD (n = 20). (F) Correlation of IL‐33 with serum CRE and serum BUN in patients with DKD. Data in (A, C, D, E) are presented as mean ± SD. ^****^ *p* < 0.0001, ^***^ *p* < 0.001, ^**^ *p* < 0.01, ^*^ *p* < 0.05; ns, not significant. Statistical significance was assessed by one‐way ANOVA with Tukey's multiple‐comparisons test.

We initially performed a comparative transcriptomic analysis using publicly available GEO datasets, profiling gene expression in db/db mice with DKD vs db/m control mice. Transcriptomic profiling identified marked upregulation of ANGPTL3 and IL‐33 mRNA in renal tissues from DKD mice relative to controls, implicating these cytokines in murine DKD pathogenesis (Figure 1A). To validate the association of these cytokines with DKD, we assessed their expression in an established murine model of the disease—db/db mice fed with high‐fat diet. Transcriptomic analysis of these samples confirmed significant activation of both ANGPTL3‐related and IL‐33/ST2 signaling pathways in the kidneys of DKD mice compared with db/m controls (Figure 1B). Furthermore, we observed a marked upregulation of both IL‐33 mRNA and protein in the renal tissues of DKD mice exhibiting compromised renal function (Figure 1C).

Subsequently, we performed differential expression analysis on transcriptomic datasets from 21 DKD patients and 9 healthy controls obtained from the GEO database. The results demonstrated significantly elevated IL‐33 expression in renal tissues from DKD patients compared to healthy controls, confirming that IL‐33 upregulation is conserved in both murine and human DKD (Figure 1D). To further elucidate the association between IL‐33 and DKD, we measured serum IL‐33 protein concentrations in 20 healthy controls and 20 DKD patients. Mirroring observations in the DKD mouse model, circulating IL‐33 concentrations were markedly elevated in patients with DKD relative to healthy controls (Figure 1E). To evaluate renal function, we measured serum creatinine (CRE) and blood urea nitrogen (BUN) as indicators of glomerular filtration and examined their relationship with circulating IL‐33 concentrations. A significant positive correlation emerged between IL‐33 concentrations and biomarkers of renal damage, implicating IL‐33 upregulation in the progression of DKD (Figure 1F).

These bioinformatic analyses collectively illustrate the significant role of both ANGPTL3 and IL‐33 signaling pathways in DKD, underscoring the potential therapeutic advantage of dual‐pathway inhibition for DKD treatment.

### 2.2 Generation and Characterization of the Bifunctional Fusion Protein Targeting both ANGPTL3 and IL‐33

**FIGURE 2:**
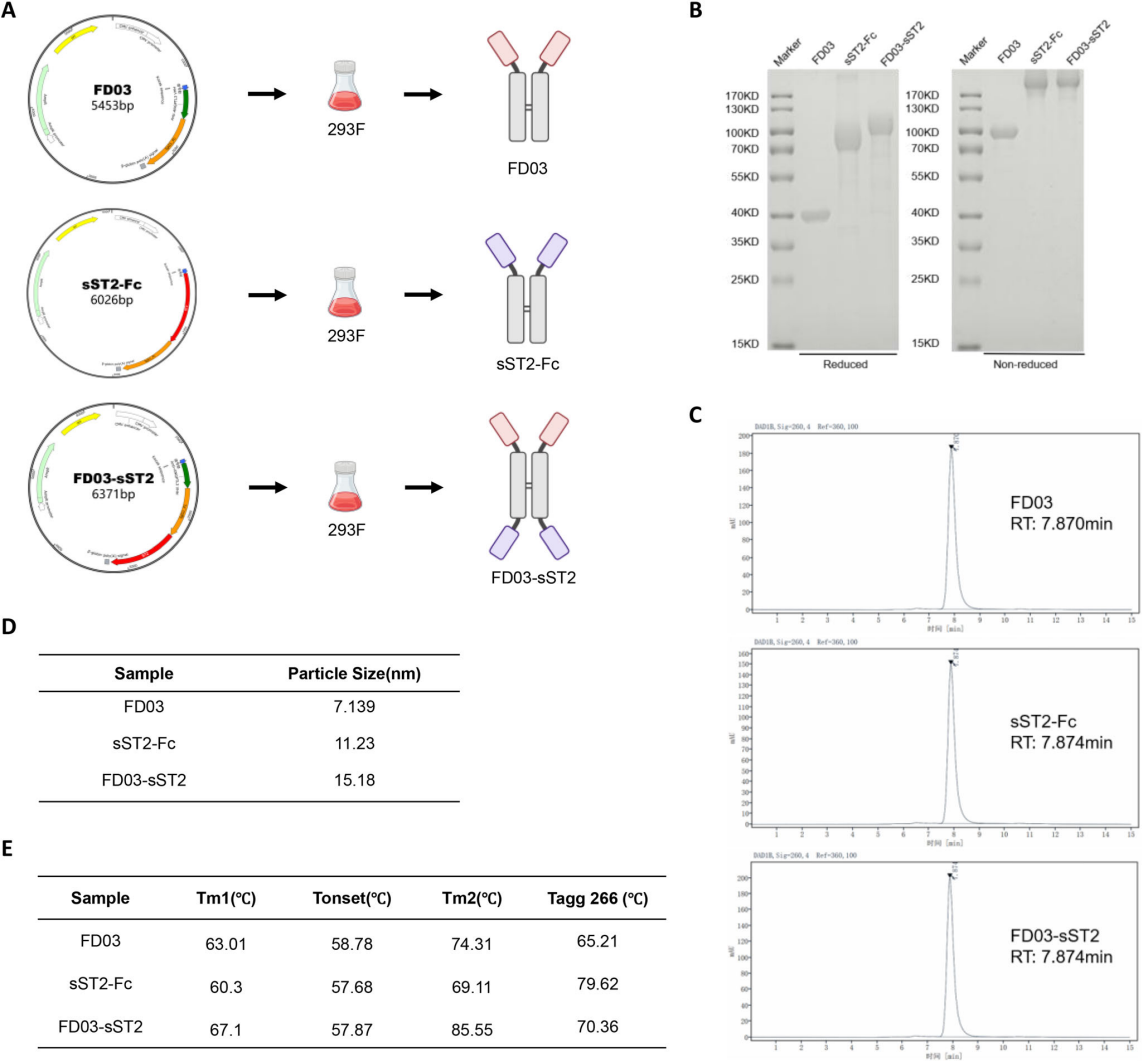
Expression, purification, and characterization of FD03‐sST2. (A) Schematic illustration of protein design, generation, and expression. (B) SDS‐PAGE of purified proteins under reducing and non‐reducing conditions. (C) SEC‐HPLC profiles of purified proteins (RT, retention time). (D) Particle size distribution of the proteins. (E) Thermal stability assessment of the proteins.

To enable concurrent targeting of ANGPTL3 and IL‐33, we constructed a bifunctional fusion protein, termed FD03‐sST2, by genetically linking FD03 to the amino terminus of a human IgG1 Fc scaffold, while sST2 was appended to the carboxyl terminus via a flexible (Gly4Ser)3Gly linker. The purity of FD03, sST2‐Fc, and FD03‐sST2 was evaluated using SDS‐PAGE under reducing and non‐reducing conditions, together with size‐exclusion chromatography (SEC‐HPLC) (Figure 2A–C). SDS‐PAGE analysis under reducing and non‐reducing conditions confirmed the expected molecular weight and high purity of FD03‐sST2, consistent with those observed for FD03 and sST2‐Fc. SEC‐HPLC analysis demonstrated high purity for all three proteins, with monomeric purity values of 100% for FD03, 100% for sST2‐Fc, and 100% for FD03‐sST2. The hydrodynamic diameters of FD03, sST2‐Fc, and FD03‐sST2 were 7.14, 11.23, and 15.18 nm, respectively, correlating with their predicted molecular weights (Figure 2D; Figure S1A). Protein stability was assessed by determining the melting temperature (Tm) and aggregation temperature (Tagg) via intrinsic protein fluorescence. FD03‐sST2 exhibited a Tm1 value of 67.1°C, indicating robust thermal stability. Based on static light scattering (SLS) at 266 nm, the Tagg value of FD03‐sST2 was 70.36°C (Figure 2E; Figure S1B). After multiple freeze‐thaw cycles, all three fusion proteins maintained >95% monomeric purity, confirming their stability under stress conditions (Figure S1C,D).

### 2.3 Characterization and Bioactivity of Bifunctional Fusion Proteins

**FIGURE 3:**
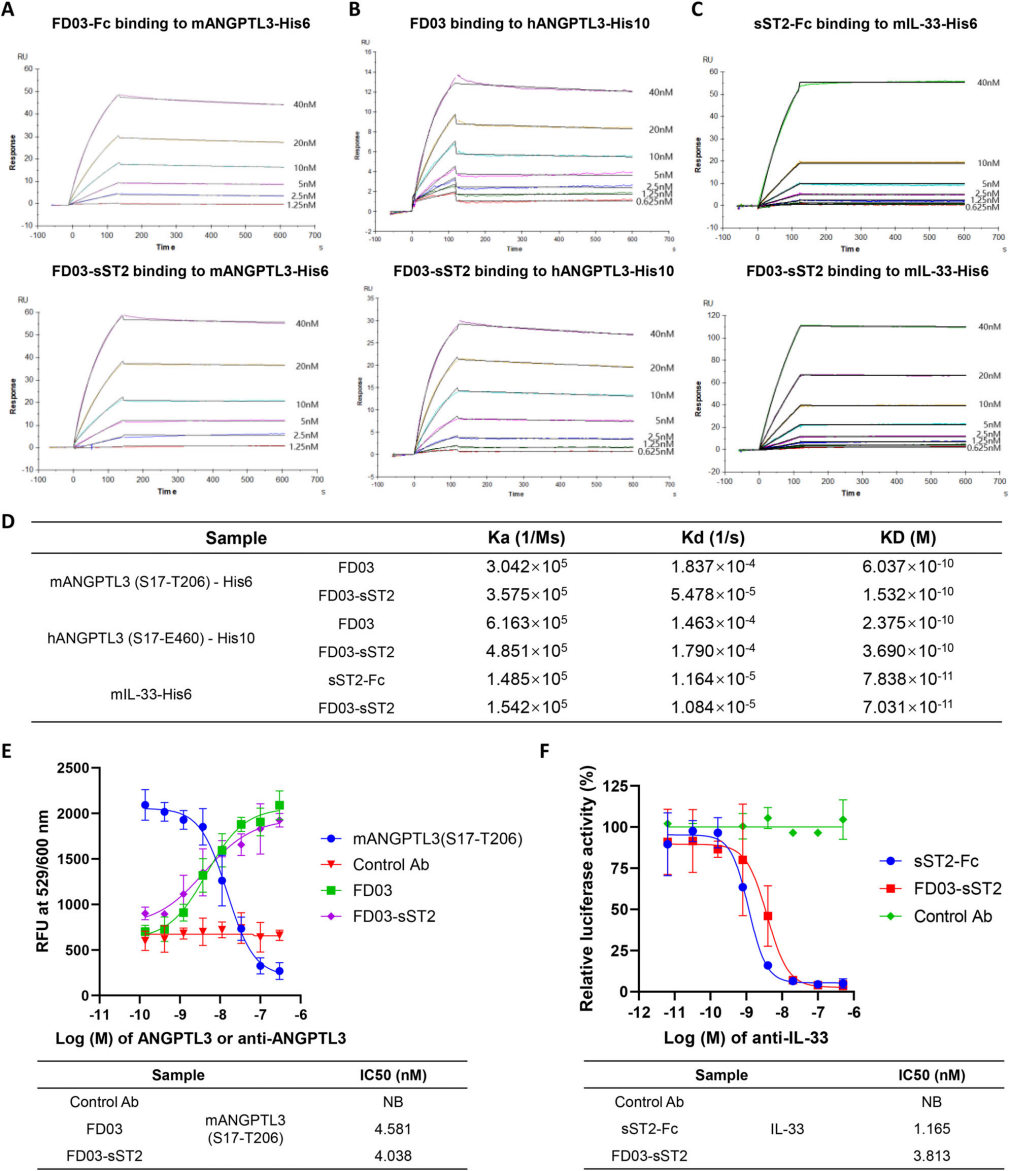
Binding affinity and bioactivity of FD03‐sST2. (A–C) Surface plasmon resonance analysis of FD03 and FD03‐sST2 binding to murine half‐length ANGPTL3 (A), human full‐length ANGPTL3 (B), and of sST2‐Fc and FD03‐sST2 to murine IL‐33 (C). (D) FD03‐sST2 exhibited ANGPTL3‐binding affinity comparable to FD03 and IL‐33‐binding affinity similar to sST2‐Fc. (E) FD03 and FD03‐sST2 counteracted ANGPTL3‐mediated LPL inhibition in a dose‐dependent manner, as determined by fluorescence intensity of a lipase substrate. (F) sST2‐Fc and FD03‐sST2 suppressed IL‐33‐stimulated luciferase reporter activity, measured by fluorescence intensity.

FD03‐sST2 exhibited high‐affinity binding to murine half‐length ANGPTL3 (KD = 0.1532 nm), human full‐length ANGPTL3 (KD = 0.3690 nm), and murine IL‐33 (KD = 0.0703 nm) (Figure 3A–C). Comparative analysis revealed that FD03‐sST2 retained binding affinities for ANGPTL3 and IL‐33 comparable to those of the parental FD03 and sST2‐Fc proteins, respectively (Figure 3D).

ANGPTL3 has been established as a physiological suppressor of LPL activity. The ability of FD03‐sST2 to counteract ANGPTL3‐mediated LPL inhibition was evaluated using an LPL activity assay. FD03 and FD03‐sST2 each restored LPL enzymatic function in a dose‐dependent fashion, overcoming the inhibition imposed by mANGPTL3(S17‐T206)‐Fc. The half‐maximal inhibitory concentrations (IC50) were determined to be 4.581 nm for FD03 and 4.038 nm for FD03‐sST2 (Figure 3E). These data confirm that both FD03 and FD03‐sST2 possess high ANGPTL3‐binding affinity and potent biological activity.

The inhibitory activity of FD03‐sST2 and sST2‐Fc against IL‐33/ST2 signaling was assessed using an NF‐κB‐driven luciferase reporter assay. The half‐maximal effective concentration (EC50) values were determined to be 0.1 nm for sST2‐Fc and 0.3 nm for FD03‐sST2 (Figure 3F).

Collectively, these results demonstrate the successful construction of FD03‐sST2, which retains potent dual‐inhibitory activity against both ANGPTL3 and IL‐33 while exhibiting favorable developability‐related properties, including high purity, thermal stability and resistance to freeze‐thaw stress. The bifunctional design therefore preserves the activities of both parental moieties within a single stable molecular scaffold, providing a practical basis for comparing single‐molecule dual targeting with the free‐drug combination in vivo.

### 2.4 FD03‐sST2 Ameliorated Renal Function and Alleviated Renal Injury in DKD Mice

**FIGURE 4:**
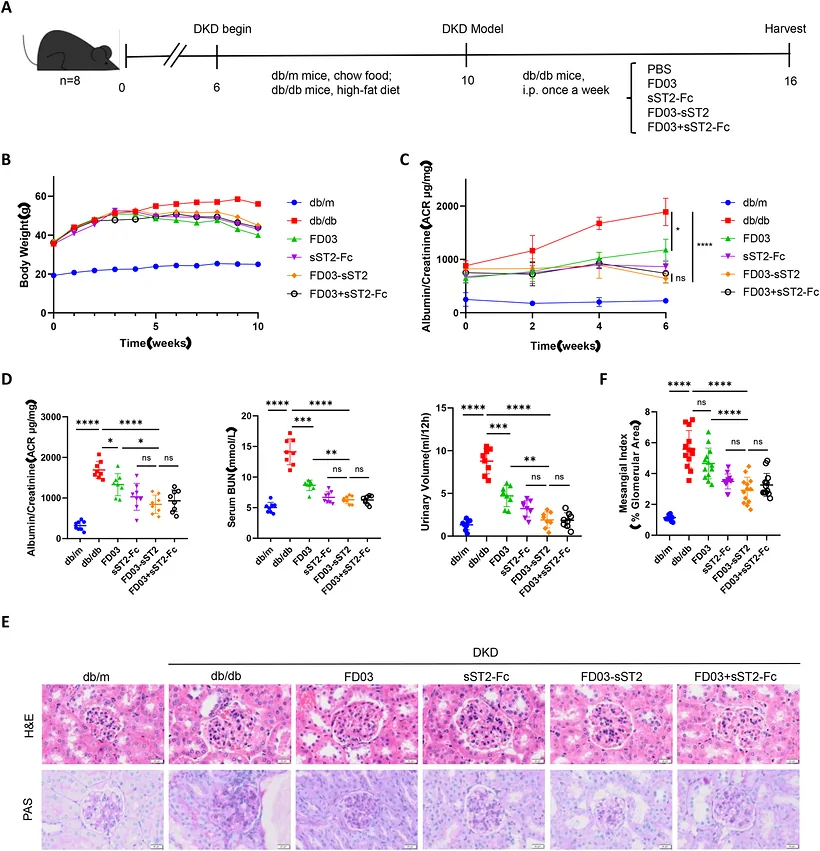
Reno protective effects of FD03‐sST2 in db/db mice: functional improvement and histopathological amelioration. (A) Experimental timeline and dosing protocol for protein administration. (B) Temporal profile of body weight changes (n = 8). (C) Longitudinal ACR measurements throughout the 6‐week intervention (n = 8). (D) ACR, serum BUN, and 12 h urine volume after 6 weeks of protein treatment in db/db mice (n = 8). (E) Representative photomicrographs of renal histology: H&E and PAS trichrome staining (scale bar = 20 µm). (F) Quantification of the glomerular mesangial index (n = 13). Data in (B, C, D, F) are presented as mean ± SD. ^****^ *p* < 0.0001, ^***^ *p* < 0.001, ^**^ *p* < 0.01, ^*^ *p* < 0.05; ns, not significant. Statistical significance was assessed by one‐way ANOVA with Tukey's multiple‐comparisons test.

To evaluate the reno protective effects of FD03‐sST2 in diabetic kidney disease, we established a DKD model by feeding db/db mice a high‐fat diet for four weeks (Figure S2A–E). Mice received treatment with the fusion protein, combination therapy, or respective monotherapies for duration of 6 weeks (Figure 4A). FD03‐sST2 treatment gradually decreased body weight and ACR, demonstrating superior efficacy to FD03 or sST2‐Fc monotherapy (Figure 4B,C). Furthermore, FD03‐sST2 markedly reduced BUN and 12 h urine volume, signifying maintained renal function (Figure 4D).

To assess renal histopathological alterations, particularly glomerular mesangial expansion (GME), we conducted hematoxylin and eosin (H&E) and periodic acid–Schiff (PAS) staining. H&E staining revealed that FD03‐sST2 treatment ameliorated renal injury more effectively than either FD03 or sST2‐Fc monotherapy (Figure 4E). FD03‐sST2 treatment significantly attenuated the marked glomerular mesangial matrix expansion and elevated mesangial index observed in db/db mice, exhibiting greater therapeutic benefit than either FD03 or sST2‐Fc alone (Figure 4E,F). Additionally, safety profiling was conducted by H&E staining of major organs from mice administered FD03‐sST2, which revealed no observable morphological damage, confirming the in vivo safety of the three fusion proteins (Figure S3).

Collectively, these data indicate that FD03‐sST2 significantly improves renal function and reduces histological damage in a mouse model of DKD, with comparable efficacy to the free‐drug combination (FD03+sST2‐Fc), supporting the non‐inferiority of the single‐molecule format.

### 2.5 FD03‐sST2 Improved Glucose and Lipid Metabolism and Reduced Hepatic and Renal Lipid Deposition

**FIGURE 5:**
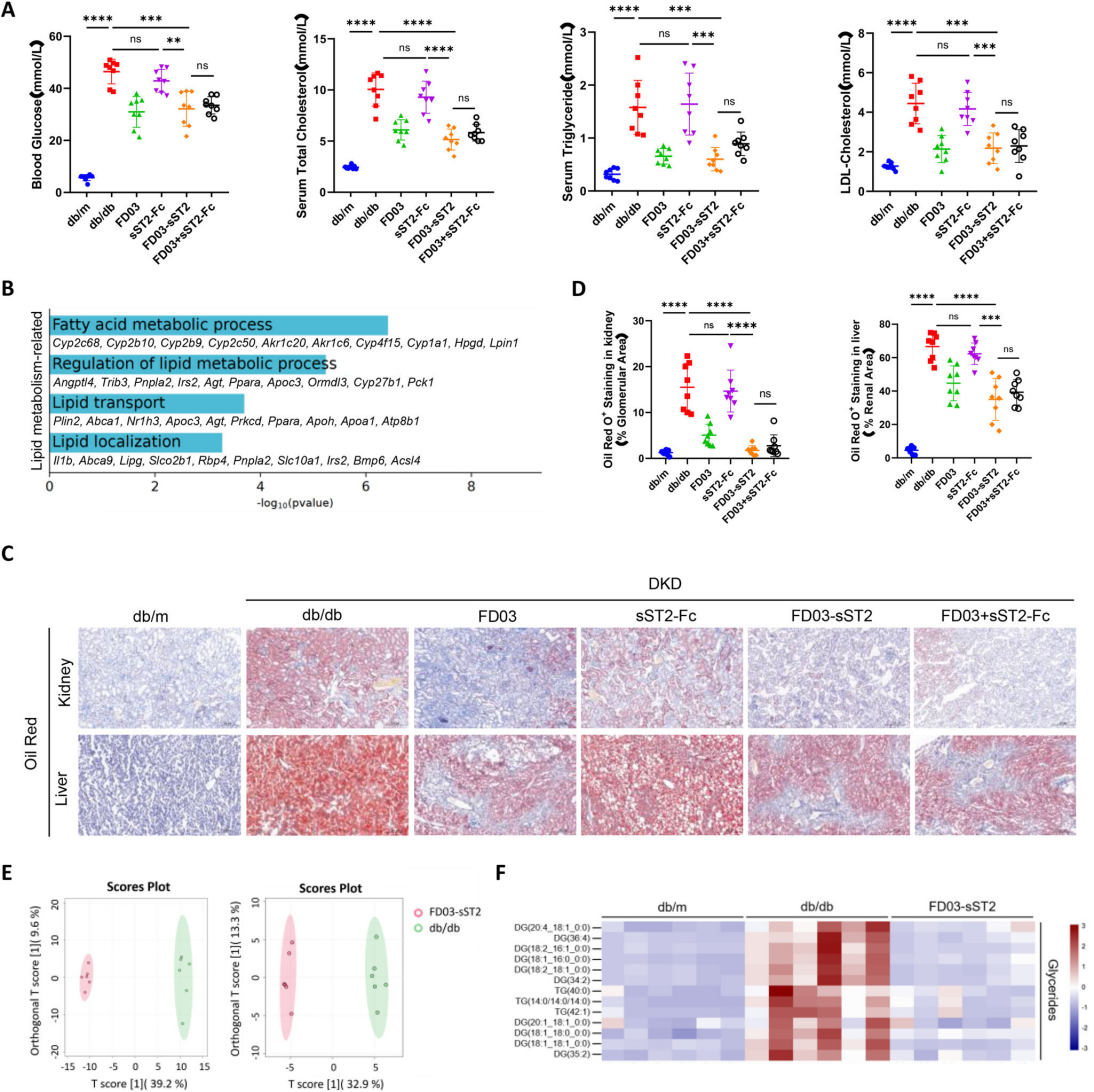
FD03‐sST2 alleviated hepatic and renal steatosis in db/db mice. (A) GLU, serum TC, serum TG, and LDL‐C after 6 weeks of protein administration (n = 8). (B) KEGG enrichment of lipid metabolism‐related genes significantly downregulated in FD03‐sST2‐treated mice. (C) Oil Red O staining of neutral lipids (NLs) in kidney and liver sections (scale bar = 200 µm). (D) Quantification of Oil Red O‐positive staining in kidney (n = 8) and liver (n = 8). (E) OPLS‐DA score plots from metabolomics analyses in BEH negative‐ion mode and C18 positive‐ion mode (n = 6). (F) Heat map of significantly altered glyceride‐related metabolites among db/m, db/db, and FD03‐sST2‐treated mice. Data in (A, D) are presented as mean ± SD. ^****^ *p* < 0.0001, ^***^ *p* < 0.001, ^**^ *p* < 0.01, ^*^ *p* < 0.05; ns, not significant. Statistical significance was assessed by one‐way ANOVA with Tukey's multiple‐comparisons test.

Research indicates that disturbances in glucose and lipid metabolism resulting from a high‐fat diet are significant contributors to the advancement of DKD [8, 38]. Consequently, we evaluated serum glucose, TG, TC, and LDL‐C levels to determine the effect of FD03‐sST2 on glucose and lipid metabolism. The findings indicated that serum glucose, TG, TC and LDL‐C levels were markedly elevated in the db/db group relative to the db/m group (Figure 5A). Prior research has indicated that inhibiting ANGPTL3‐mediated lipase suppression substantially decreases free cholesterol and LDL‐C levels in the bloodstream [17, 39], thereby dramatically mitigating lipotoxicity and enhancing renal function in DKD murine models. In our investigation, the heightened markers in db/db mice were mitigated following medication intervention, and analogous hypoglycemia and hypolipidemic effects were noted in the FD03‐sST2, combination and FD03 treatment groups. Subsequently, according to Kyoto Encyclopedia of Genes and Genomes (KEGG) analysis, compared with the db/db group, the lipid metabolism‐related signaling pathways that exhibited significant alterations following FD03‐sST2 administration, mainly involved fatty acid metabolic process (KEGG: mmu00071, KEGG: mmu01212), regulation of lipid metabolic process (KEGG: mmu00564, KEGG: mmu04975), lipid transport (KEGG: mmu04979), lipid localization (KEGG: mmu04975, KEGG: mmu04146), etc., indicating that FD03‐sST2 may facilitate renal repair by modulating lipid metabolism disorders (Figure 5B).

Lipid droplet accumulation in DKD patients and experimental models is associated with renal impairment [40, 41]. To assess whether FD03‐sST2 reduces hepatic and renal lipid accumulation, we evaluated neutral lipids by Oil Red O staining. Tissue sections demonstrated a substantial elevation of NLs in db/db mice, which was dramatically reduced by FD03‐sST2, combination therapy, and FD03 monotherapy (Figure 5C). Notably, quantitative analysis of Oil Red O‐positive regions revealed the most significant decrease in lipid accumulation in the FD03‐sST2‐treated group (Figure 5D). Metabolomic profiling of kidney tissue was conducted using PCA based on metabolite concentrations (MetaboAnalyst), revealing distinct separation between the FD03‐sST2 group and the db/db group, signifying a transition in renal metabolic phenotype (Figure 5E). Subsequent focused examination demonstrated that FD03‐sST2 markedly reduced renal concentrations of TG and diacylglycerols (DG), so substantially ameliorating renal lipid metabolism toward levels observed in control mice (Figure 5F).

The FD03 moiety in the bifunctional protein maintains its ability to improve glucose and lipid metabolic disorders and decrease tissue lipid buildup.

### 2.6 FD03‐sST2 Alleviated Renal Inflammation in db/db Mice by Inhibiting the NF‐κB/NLRP3 Pathway

**FIGURE 6:**
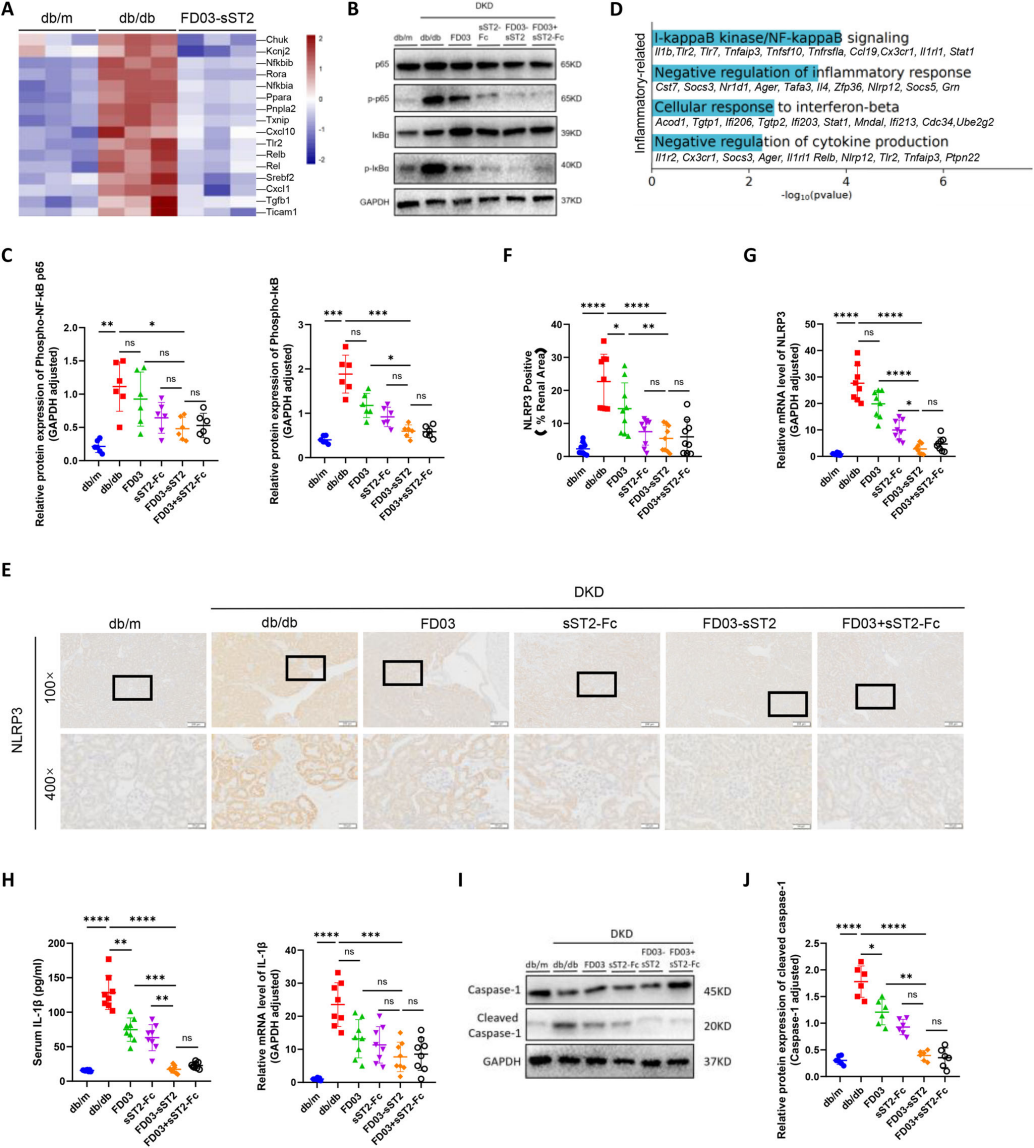
Modulation of the NF‐κB/NLRP3 inflammasome pathway by FD03‐sST2 in db/db mice. (A) Heat map of significantly altered inflammation‐related metabolites among db/m, db/db, and FD03‐sST2‐treated mice. (B, C) Western blot analysis of p65 and IκBα in renal lysates post‐treatment; GAPDH was used as an internal loading reference (n = 6). (D) KEGG enrichment of inflammation‐related genes significantly downregulated in FD03‐sST2‐treated mice. (E) Representative immunohistochemical images of NLRP3 in renal sections at 100× and 400× magnification; scale bars: 200 µm (100×) and 50 µm (400×). (F) Quantification of NLRP3 staining in kidney sections (n = 9). (G) Renal *Nlrp3* mRNA expression after protein administration (n = 8). (H) Serum IL‐1β levels and renal *Il1b* mRNA expression after protein administration (n = 8). (I, J) Western blot analysis of caspase‐1 and its cleaved form in renal lysates following treatment (n = 6). Data in (C, F, G, H, J) are presented as mean ± SD. ^****^ *p* < 0.0001, ^***^ *p* < 0.001, ^**^ *p* < 0.01, ^*^ *p* < 0.05; ns, not significant. Statistical significance was assessed by one‐way ANOVA with Tukey's multiple‐comparisons test.

High levels of proinflammatory cytokines correlate with DKD progression [42, 43]. KEGG analysis revealed that, in comparison to the db/db group, the inflammatory‐related signaling pathways that were significantly modified following FD03‐sST2 administration primarily encompassed IκB kinase/NF‐κB signaling (KEGG: mmu04064), negative regulation of inflammatory response (KEGG: mmu04620, KEGG: mmu04621), cellular response to interferon‐beta (KEGG: mmu04630), and negative regulation of cytokine production (KEGG: mmu04630, KEGG: mmu04060), etc., indicating that FD03‐sST2 may facilitate renal repair by attenuating inflammatory responses (Figure 6D). Consequently, we examined the anti‐inflammatory efficacy of bifunctional fusion protein FD03‐sST2 treatment in preserving renal function.

Elevated glucose levels may stimulate NF‐κB phosphorylation, consequently activating the transcription of the NLRP3 inflammasome, which has been shown to rise in DKD and contribute to renal injury [39, 44]. Simultaneously, IL‐33, elevated as a result of renal damage, interacts with ST2L on cells to initiate an NF κB‐dependent signaling cascade, primarily enhancing the transcriptional expression of NLRP3 inflammasome constituents and its principal substrate pro‐IL‐1β [36, 45]. Volcano plot analysis revealed 2195 significantly modified genes in the FD03‐sST2‐treated group compared to the db/db group. Cluster analysis indicated that genes related to the NF‐κB pathway and inflammatory response were markedly downregulated following FD03‐sST2 treatment (Figure 6A). Additionally, we observed that NF‐κB p65 protein expression and NF‐κB IκB protein expression were decreased following FD03‐sST2 treatment. These results indicate that concurrent inhibition of ANGPTL3 and IL‐33 in db/db mice modulates NF‐κB signaling, implicating this pathway in the pathogenesis of DKD‐associated renal injury (Figure 6B,C).

The administration of FD03‐sST2 markedly diminished the positive staining area of NLRP3 in kidney sections relative to the db/db group and those treated with FD03 or sST2‐Fc (Figure 6E,F). NLRP3 mRNA levels were significantly reduced by FD03‐sST2 treatment relative to the db/db group, with the extent of downregulation exceeding that achieved by either FD03 or sST2‐Fc treatment (Figure 6G). In contrast, the activation of cleaved caspase‐1 and the transcription and production of IL‐1β were markedly diminished following FD03‐sST2 therapy compared to the db/db group, which was also lower than the FD03 or sST2‐Fc treatments (Figure 6H–J). These results demonstrate that FD03‐sST2 suppresses NLRP3 inflammasome activation and inflammatory mediator synthesis.

In conclusion, FD03‐sST2 attenuates renal inflammation and preserves renal function in DKD mice by potently blocking the NF‐κB pathway, mitigating NLRP3 activation, and downregulating inflammatory factors.

### 2.7 FD03‐sST2 Inhibited the IL‐33/ST2/ILC2 Signal Transduction Pathway, Thereby Reducing Renal Fibrosis in db/db Mice

**FIGURE 7:**
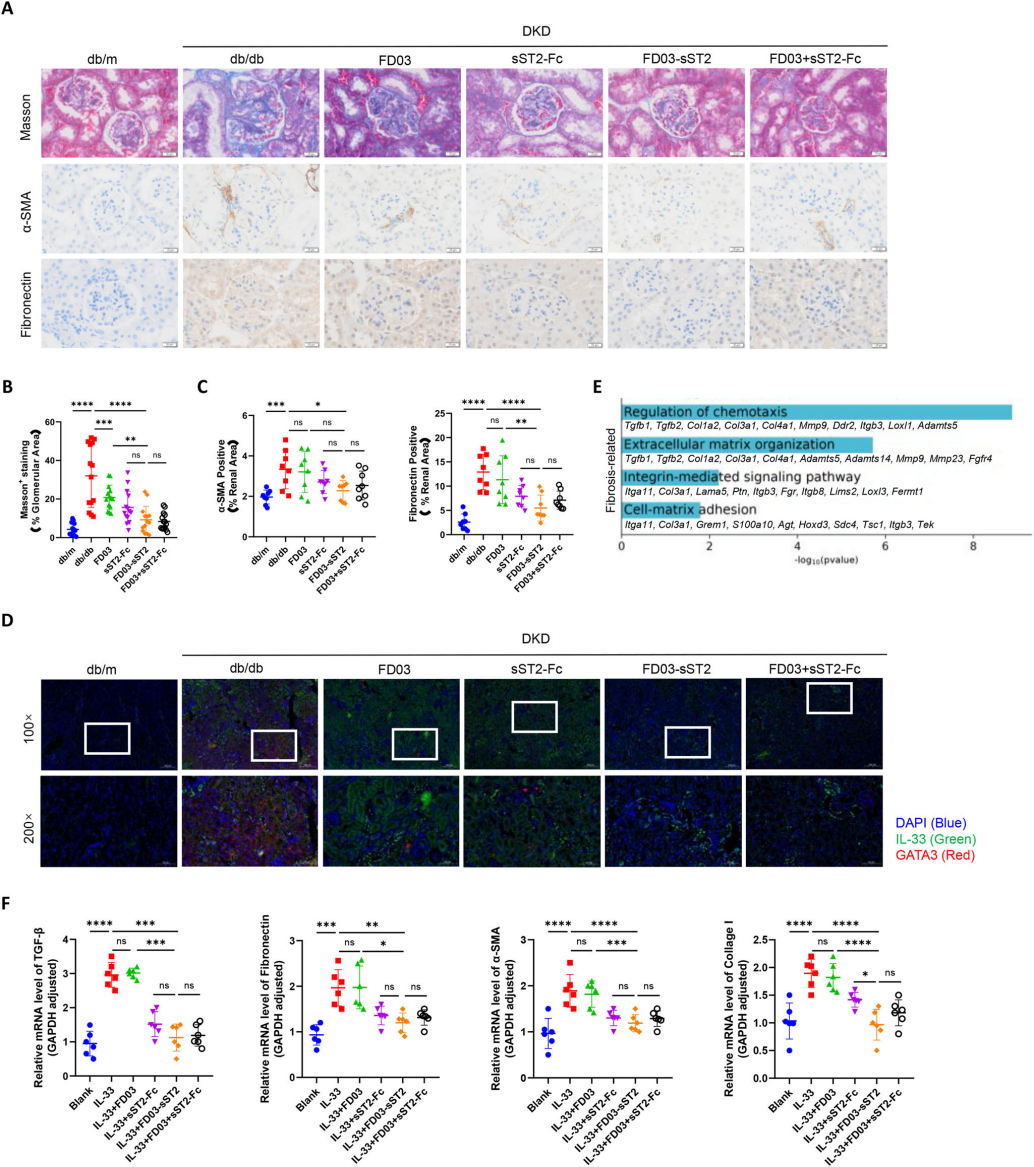
FD03‐sST2 mitigates renal fibrosis in db/db mice through suppression of ILC2‐derived fibrosis‐related factors (A) Representative images of kidney sections: Masson's trichrome (scale bar = 20 µm) and immunohistochemical staining ofα‐SMA and fibronectin (scale bar = 20 µm). (B) Quantitative analysis of collagen deposition based on Masson‐stained sections (n = 14). (C) Quantitative assessment of renal α‐SMA (n = 8) and fibronectin (n = 8) levels following treatment. (D) Representative immunofluorescence micrographs of db/db mouse kidneys (n = 8) showing DAPI (blue), IL‐33 (green), and GATA3 (red). Magnification: 100× and 400×; scale bars: 200 µm (100×) and 100 µm (400×). (E) KEGG enrichment of fibrosis‐related genes significantly downregulated in FD03‐sST2‐treated mice. (F) Transcript levels of TGF‐β, fibronectin, α‐SMA, and collagen I in cultured ILC2s (n = 6). Data in (B, C, F) are presented as mean ± SD. ^****^ *p* < 0.0001, ^***^ *p* < 0.001, ^**^ *p* < 0.01, ^*^ *p* < 0.05; ns, not significant. Statistical significance was assessed by one‐way ANOVA with Tukey's multiple‐comparisons test.

Given the close association between renal function and fibrotic progression in DKD [46], we next examined the anti‐fibrotic potential of FD03‐sST2 in db/db mice. The extent of renal fibrosis was evaluated by Masson's trichrome staining of kidney sections. Masson staining indicated considerable renal collagen deposition in db/db animals, which markedly diminished following treatment with the fusion proteins (Figure 7A). Quantification of Masson‐positive areas revealed significantly reduced collagen deposition in the FD03‐sST2‐treated group (Figure 7B). Furthermore, immunohistochemical analysis of fibronectin and α‐smooth muscle actin (α‐SMA) expression was conducted to determine the effect of FD03‐sST2 on fibrotic progression. In db/db mice, renal fibronectin and α‐SMA levels were significantly elevated compared to normal controls (Figure 7A). FD03‐sST2 administration suppressed these fibrotic markers, with a more pronounced effect on fibronectin, and ameliorated glomerular and interstitial fibrosis (Figure 7C). Together, these findings demonstrate that FD03‐sST2 effectively attenuates renal fibrosis in db/db mice.

As pivotal effector cells of IL‐33, ILC2s are activated by IL‐33 to secrete pro‐fibrotic factors [20, 31]. Immunofluorescence labeling results for IL‐33 and GATA3 (a marker for ILC2s) in DKD mice kidneys indicated that IL‐33 levels in kidney tissue of db/db mice were significantly raised compared to normal animals, alongside a notable increase in activated ILC2 cells (Figure 7D). FD03‐sST2 reduced renal IL‐33 and GATA3 signals in DKD kidneys, supporting an in vivo association between IL‐33 upregulation and ILC2 activation that was attenuated by treatment. To further test whether the IL‐33/ST2/ILC2 axis is functionally linked to renal fibrosis, transcriptome sequencing was performed on renal tissues and complemented by ex vivo stimulation experiments using isolated renal ILC2s. KEGG pathway enrichment analysis showed that FD03‐sST2 predominantly modulated pathways related to chemotaxis (KEGG: mmu04062), extracellular matrix organization (KEGG: mmu04512, KEGG: mmu04151), integrin‐mediated signaling pathway (KEGG: mmu04510, KEGG: mmu04810), cell‐matrix adhesion(KEGG: mmu04510, KEGG: mmu04512), etc., consistent with attenuation of a profibrotic transcriptional program downstream of IL‐33‐responsive immune activation (Figure 7E). To directly examine whether IL‐33 can instruct ILC2s toward a profibrotic output at the level of gene expression, renal ILC2s isolated from normal mice were stimulated ex vivo with IL‐33 and the transcript levels of the profibrotic mediators TGF‐β, fibronectin, α‐SMA and collagen I were measured. This reductionist approach, by focusing specifically on purified ILC2s rather than bulk renal tissue, provides a cell‐type‐resolved functional readout that circumvents the dilution effect inherent to whole‐organ transcriptomics, in which ILC2s represent a minor cell fraction (<1% of renal immune cells). IL‐33 stimulation significantly upregulated all four profibrotic transcripts compared with unstimulated controls, directly demonstrating that IL‐33 is sufficient to drive a fibrosis‐related transcriptional program in renal ILC2s (Figure 7F). FD03‐sST2 markedly reduced these profibrotic factors, demonstrating that the fusion protein directly interrupts the IL‐33‐driven profibrotic response in ILC2s at the transcriptional level. FD03‐sST2 markedly reduced these profibrotic factors, demonstrating that the fusion protein directly interrupts the IL‐33‐driven profibrotic response in ILC2s. The magnitude of inhibition was comparable to that of sST2‐Fc and the free‐drug combination in this reductionist assay.

Together, these data support a model in which renal IL‐33 promotes fibrosis, at least in part, by driving a profibrotic ILC2 program, and FD03‐sST2 mitigates renal fibrosis by interrupting this IL‐33/ST2/ILC2 effector axis.

## 3 Discussion

DKD persists as a major cause of ESRD globally, arising from a complex network of metabolic dysfunction, chronic inflammation, and advancing fibrosis [3, 47]. Notwithstanding improvements in glycemic, cholesterol, and blood pressure management, a considerable number of individuals continue to experience disease progression, highlighting the necessity for medicines that address the fundamental pathogenic networks [6, 8]. A considerable unmet need persists for therapies that simultaneously target the interconnected metabolic and inflammatory factors contributing to the pathogenesis of DKD [4, 48]. Consequently, creating innovative therapies that can simultaneously target these fundamental pathogenic pathways is essential for enhancing clinical results. This work presents FD03‐sST2, a bifunctional fusion protein capable of dual targeting ANGPTL3 and IL‐33. Treatment with this agent in a murine DKD model substantially improved renal function and mitigated inflammation and fibrosis, primarily by ameliorating systemic lipid abnormalities and intercepting the maladaptive IL‐33‐driven inflammatory‐fibrotic axis. These data suggest a unified mechanistic model in which lipid poisoning triggers renal damage and IL‐33 release, subsequently sustaining harm through NF‐κB/NLRP3 activation and ILC2‐mediated fibrosis. FD03‐sST2 disrupts this vicious cycle at two critical nodes, and its bifunctional single‐molecule design provides a translationally attractive way to co‐target these nodes in a coordinated manner.

Dysregulation of lipid metabolism and subsequent ectopic lipid accumulation in the kidney are key drivers of DKD pathogenesis [4, 8]. Consistent with this idea, our transcriptome investigations of db/db mice demonstrated considerable enrichment of pathways related to fatty acid transport and lipid metabolism. Specifically, we identified ANGPTL3 as a key upregulated regulator. ANGPTL3 suppresses LPL function, resulting in increased circulating TG and TC that promote lipid accumulation in peripheral tissues, including the kidney. Accordingly, db/db mice exhibited significant renal lipid accumulation accompanied by functional decline. The FD03 moiety of our fusion protein, an anti‐ANGPTL3 nanobody, successfully reinstated LPL activity, as verified by in vitro assays. In vivo, FD03‐sST2 administration markedly decreased serum TG, TC and LDL‐C levels while simultaneously reducing renal lipid accumulation. The enhancement in systemic and renal lipid balance correlated with a significant improvement in essential renal function metrics, such as urine ACR and BUN. These findings highlight that rectifying ANGPTL3‐mediated lipid accumulation alleviates lipotoxic stress on renal cells, establishing a critical foundation for renoprotection.

**FIGURE 8:**
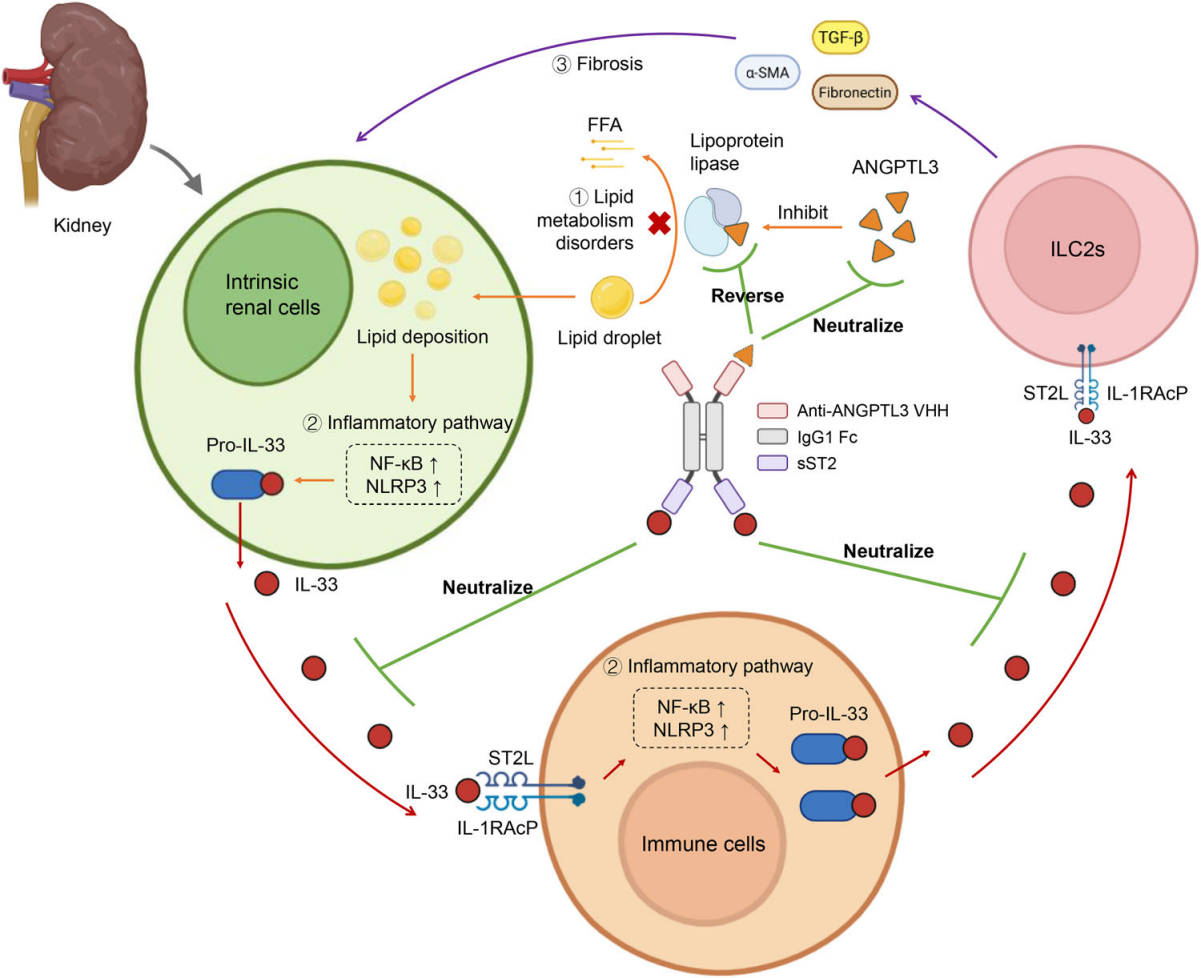
Proposed model of DKD progression and the role of FD03‐sST2 in improving dysregulated lipid metabolism, alleviating inflammation, and inhibiting fibrosis.

In addition to its direct cellular toxicity, lipid excess acts as a significant catalyst for sterile inflammation, a characteristic feature of DKD pathophysiology [9, 42]. Our research outlines a distinct mechanism connecting lipotoxicity to a prolonged inflammatory response mediated by the alarmin IL‐33 [49, 50]. Consistent with this concept, treatment of renal tubular cells (TCMK‐1 and HK‐2) with palmitic acid resulted in a dose‐dependent upregulation of Il33 mRNA and increased IL‐33 protein secretion, directly demonstrating that lipid overload is a sufficient upstream stimulus for IL‐33 release in renal parenchymal cells (Figure S4A). IL‐33, secreted by injured epithelium and endothelial cells, serves as a powerful activator of the innate immune system through its receptor ST2L. We discovered that IL‐33/ST2 signaling was excessively active in DKD, resulting in subsequent NF‐κB activation. Consistent with these findings, we observed enhanced nuclear translocation of NF‐κB p65 and elevated expression of pro‐inflammatory cytokines (TNF‐α, IL‐1β, IL‐6). Given that NF‐κB signaling promotes NLRP3 inflammasome activation via transcriptional upregulation of its components, we next examined inflammasome status in our model. Indeed, increased NLRP3 expression, caspase‐1 cleavage, and mature IL‐1β secretion confirmed augmented inflammasome assembly and activation. This establishes a self‐perpetuating cycle: initial lipid‐induced injury releases IL‐33, which fuels NF‐κB/NLRP3‐dependent inflammation, leading to further tissue damage and additional IL‐33 release. FD03‐sST2 disrupted this circuit at two locations. The FD03 moiety mitigated kidney damage in the initial phases of DKD by enhancing lipid metabolism and decreasing the release of the alarm factor IL‐33. Second, the sST2 decoy receptor competes with membrane‐bound ST2L for IL‐33 binding, thereby attenuating NF‐κB and NLRP3 activation (Figure 8). The combined pharmacology embodied by FD03‐sST2 produced better control of renal inflammation than either monotherapy, supporting the therapeutic value of simultaneously targeting both axes.

Prolonged IL‐33 signaling in the kidney drives ILC2 activation, prompting these cells to release type 2 cytokines and profibrotic factors, including TGF‐β [29, 30]. It is noteworthy that although bulk renal RNA‐seq did not reveal statistically significant enrichment of TGF‐β/Smad or integrin signaling pathways at the whole‐tissue level, this is consistent with the biology of ILC2s as a numerically rare cell population that constitutes a minor fraction of total renal cells. The transcriptional output of ILC2s is likely diluted within the heterogeneous cellular composition captured by bulk sequencing, rendering pathway‐level enrichment statistically underpowered in this context. Critically, the ex vivo stimulation experiment using purified renal ILC2s (Figure 7F) provides direct, cell‐type‐resolved evidence that IL‐33 is sufficient to transcriptionally induce a profibrotic program—including TGF‐β, fibronectin, α‐SMA, and collagen I—in these cells, and that FD03‐sST2 effectively suppresses this program. Future studies employing single‐cell RNA sequencing or transcriptomic profiling of flow‐sorted renal ILC2s will be valuable to further characterize the cell‐intrinsic transcriptional changes within this population during DKD progression and treatment. Our immunofluorescence study demonstrated a substantial elevation of GATA3^+^ ILC2s co‐localizing with regions of elevated IL‐33 expression in DKD kidneys. Activated ILC2s were a primary source of TGF‐β, a master regulator that promotes myofibroblast development and extracellular matrix (ECM) production. Consequently, db/db animals demonstrated pronounced renal fibrosis, characterized by extensive collagen accumulation and heightened expression of fibronectin and α‐SMA. FD03‐sST2, via its sST2 domain, neutralized IL‐33, thus inhibiting ILC2 activation and curtailing TGF‐β production. This resulted in a significant decrease in ECM buildup, as demonstrated by Masson's staining and immunohistochemistry. Transcriptomic research corroborated this approach, indicating that FD03‐sST2 treatment downregulated genes involved in ECM organization, integrin signaling and cell‐matrix adhesion pathways that were upregulated in DKD. By inhibiting the IL‐33/ILC2 pathway, FD03‐sST2 directly targeted the fibrotic endpoint of DKD progression.

The synergistic effectiveness of FD03‐sST2 arises from its comprehensive design, which simultaneously targets the metabolic origins and the inflammatory/fibrotic consequences of DKD. The FD03 arm alleviates the upstream cause of lipid metabolic dysfunction, hence diminishing the stimulus for IL‐33 release and ensuing inflammation. In contrast, the sST2 arm intercepts the downstream alarmin, inhibiting its ability to initiate and sustain the NF‐κB/NLRP3 inflammatory response and ILC2‐driven fibrosis. These two mechanisms are interrelated and mutually reinforcing. Mitigating inflammation and fibrosis likely enhances renal hemodynamics and cellular vitality, hence indirectly facilitating improved metabolic management. Conversely, enhancing the metabolic environment alleviates the impact of inflammatory stimuli. This bidirectional pathogenic coupling helps explain why a bifunctional strategy can outperform monotherapies in ameliorating glomerular histology and overall renal function. It is noteworthy that the in vivo efficacy of FD03‐sST2 was statistically comparable to that of the free‐drug combination (FD03+sST2‐Fc) across most endpoints, including ACR, serum lipids, and histological fibrosis scores (Figures 4, 5, 6, 7). While this indicates non‐inferiority rather than superiority of the fusion format under the current experimental conditions, the bifunctional design confers several translational advantages that are independent of raw efficacy: (i) fixed‐stoichiometry co‐delivery ensures that both therapeutic activities are always present in a defined molar ratio, eliminating the pharmacokinetic variability inherent to administering two separate molecules with potentially different half‐lives and tissue distributions; (ii) a single molecular entity simplifies the regulatory and manufacturing pathway compared with a two‐drug combination regimen; and (iii) the Fc‐mediated FcRn recycling mechanism and the hydrodynamic diameter of 15.18 nm collectively support extended systemic exposure and reduced renal clearance for the single fusion protein. These conceptual advantages, particularly fixed stoichiometry and reduced complexity, represent meaningful translational benefits that justify the development of the bifunctional format.

Our transcriptome results offer a comprehensive validation of this treatment approach. RNA sequencing of renal tissues from db/db mice demonstrated extensive dysregulation of genes associated with lipid metabolism, inflammatory response and fibrotic signaling. FD03‐sST2 therapy substantially reversed a large proportion of these transcriptional alterations, especially those associated with the recognized pathogenic pathways. The pathway enrichment analysis of the reversed gene collection validated substantial normalization of lipid metabolic processes, NF‐κB signaling, cytokine‐mediated pathways and ECM‐related biological functions. The alterations in gene expression validate the behavioral enhancements and confirm that FD03‐sST2 reinstates homeostatic transcriptional pathways disrupted in DKD.

This therapeutic strategy offers distinct advantages within the current treatment landscape. The choice of sST2 as the IL‐33‐neutralizing moiety, rather than a conventional anti‐IL‐33 monoclonal antibody, was deliberate. As an endogenous decoy receptor, sST2 operates through a mechanism that is already present in normal physiology, potentially offering a more balanced modulation of the IL‐33/ST2 axis. By sequestering free IL‐33 without directly interfering with the ST2L receptor itself, sST2 preserves the receptor's availability for any residual protective IL‐33 signaling (e.g., in tissue repair contexts), while still effectively blocking the pathological IL‐33 amplification loop observed in DKD. This mechanistic nuance distinguishes the sST2‐based approach from complete receptor blockade strategies and may contribute to the favorable safety profile observed in the organ histology assessments (Figure S3). SGLT2 inhibitors and GLP‐1 receptor agonists confer renal advantages mostly via glycemic and hemodynamic mechanisms [6], whereas FD03‐sST2 distinctively integrates robust lipid‐lowering with targeted immunomodulation of the IL‐33 alarmin pathway. IL‐33 levels are particularly pertinent as they correlate with the course of DKD progression in people [27] and ANGPTL3 suppression is an established method for managing dyslipidemia [17]. The bifunctional fusion protein format ensures concurrent target engagement and fixed‐stoichiometry co‐delivery within a single molecule, which may simplify dosing and reduce the variability inherent to administering two separate biologics. From a pharmacokinetic perspective, the IgG1 Fc scaffold incorporated into FD03‐sST2 is expected to confer an extended serum half‐life through FcRn‐mediated recycling, analogous to other Fc‐fusion proteins that typically exhibit half‐lives of 7–14 days in rodents and longer in humans [51]. Furthermore, the hydrodynamic diameter of FD03‐sST2 (15.18 nm) substantially exceeds the renal glomerular filtration threshold of approximately 8 nm, supporting its retention in the systemic circulation and reducing urinary clearance. The sustained in vitro serum stability (>95% monomeric purity over 48 h; Figure S1C) further corroborates the molecule's physicochemical integrity under physiological conditions. Together, these properties provide a theoretical basis for the once‐weekly intraperitoneal dosing regimen employed in this study, although direct in vivo pharmacokinetic profiling will be required to fully characterize systemic exposure and tissue distribution in future studies. Notably, in the ex vivo ILC2 assay, FD03‐sST2 inhibited IL‐33‐induced profibrotic factor expression to an extent similar to sST2‐Fc and the free‐drug combination, indicating that the principal added value of the fusion format in the current study is not stronger intrinsic IL‐33 neutralization per se, but rather integration of two complementary activities into one developable therapeutic molecule. Importantly, FD03‐sST2 did not induce overt histological toxicity in major organs, supporting the feasibility of this bifunctional format for further development. Our mechanistic data support an IL‐33–ILC2 profibrotic axis in DKD, but they do not yet fully exclude contributions from other IL‐33‐responsive cell populations; therefore, future studies using ILC2 depletion or lineage‐tracing approaches will be valuable to further strengthen causality.

Optimal glycemic control reduces DKD risk but does not halt disease progression, as multiple factors beyond hyperglycemia contribute to its pathogenesis. This underscores the need for therapies targeting these additional drivers, the central objective of this study. FD03‐sST2 effectively ameliorated lipid abnormalities and their inflammatory and fibrotic consequences. The observed reduction in blood glucose levels (Figure 5A) likely reflects an indirect effect of improved lipid metabolism: by restoring LPL activity and reducing ectopic lipid deposition, FD03‐sST2 may partially alleviate lipid‐induced insulin resistance, thereby contributing to modest glycemic improvement (Figure S4B and C). Additionally, we monitored food intake and found no significant difference between treatment groups, reinforcing the above conclusion that the observed metabolic improvements are attributable to the pharmacological actions of FD03‐sST2 rather than secondary to changes in caloric intake (Figure S2F). However, the renoprotective effects of FD03‐sST2 are unlikely to be solely dependent on this glycemic improvement, as the direct suppression of the NF‐κB/NLRP3 inflammatory cascade and the IL‐33/ILC2 fibrotic axis represents a glucose‐independent mechanism of renal protection—analogous to the glucose‐independent renoprotective mechanisms described for SGLT2 inhibitors [52]. Detailed glycemic profiling, including assessment of insulin sensitivity, hepatic gluconeogenesis, and muscle glucose uptake, as well as investigation of synergistic potential with established anti‐diabetic agents (e.g., SGLT2 inhibitors, GLP‐1 receptor agonists), remains an important direction for future research.

This study elucidates a DKD pathogenic cascade in which lipotoxicity triggers renal injury and IL‐33 release, amplifying damage through NF‐κB/NLRP3‐driven inflammation and ILC2‐mediated fibrosis. We demonstrate that FD03‐sST2 disrupts this cycle by simultaneously inhibiting ANGPTL3 and IL‐33. By ameliorating lipid accumulation, suppressing inflammation, and attenuating fibrosis, FD03‐sST2 preserves renal structure and function. Collectively, these findings delineate a mechanistic framework for DKD pathogenesis and establish dual ANGPTL3/IL‐33 inhibition as a promising therapeutic strategy. FD03‐sST2 thus represents a novel, translatable candidate for clinical investigation.

## 4 Materials and Methods

### 4.1 Vector Construction, Protein Expression and Purification

Three recombinant fusion proteins were generated. The FD03 construct was created by fusing the cDNA encoding an in‐house anti‐ANGPTL3 nanobody to the human IgG1 Fc region. The sST2‐Fc expression cassette was generated by joining the murine sST2 coding sequence to the IgG1 Fc domain derived from FD03. For FD03‐sST2, the sST2 cDNA was appended to the C‐terminus of FD03 via a flexible (Gly4Ser)3Gly linker. These genetic sequences were subcloned into pTT5 plasmids utilizing EcoRI and BamHI restriction sites (TaKaRa, Shiga, Japan). Subsequent protein synthesis was executed in HEK293F cells through transient transfection protocols. Following a 7‐day incubation, the extracellular fractions were collected, and the target candidates were isolated using Protein A‐based affinity chromatography (GE Healthcare, Piscataway, USA).

### 4.2 Cell Culture

Expi293F cells (iCareab Biotechnology, Suzhou, China) were maintained in a 1:1 mixture of SMM 293‐TII complete medium (Sino Biological, Beijing, China) and OPM‐293 CD05 medium (Opmbiosciences, Shanghai, China). Cultures were agitated continuously at 120 rpm with a 50 mm amplitude on a shaker (Thermo Fisher Scientific, Uppsala, Sweden).

### 4.3 Sec‐Hplc

To determine the molecular homogeneity of FD03, sST2‐Fc, and FD03‐sST2, SEC‐HPLC analysis was performed using a TSKgel G3000WXL column (7.8 mm × 30 cm, 5 µm; TOSOH) integrated into an Agilent 1260 Infinity II SFC platform. Isocratic separation was achieved with phosphate‐buffered saline (PBS) serving as the mobile phase, maintaining a constant flow of 1.0 mL/min. For each 100 µg protein injection, the eluate was monitored via dual‐wavelength UV detection (260 and 280 nm). The entire chromatographic process was executed at a controlled temperature of 37°C over a 30 min duration.

### 4.4 Serum Stability Analysis

FD03‐sST2 was incubated in filter‐sterilized serum from C57BL/6J mice at 37°C in a CO_2_ incubator for 0.01, 6, 12, 24, and 48 h. At each time point, aliquots were collected to assess residual in vitro bioactivity.

### 4.5 Thermal Stability Analysis

The conformational stability of the proteins was quantified by evaluating their melting temperatures (Tm​) on an UNcle platform. To monitor the exposure of hydrophobic clusters during unfolding, SYPRO Orange (Thermo Fisher Scientific, Uppsala, Sweden) was incorporated to facilitate fluorescence signal acquisition at 473 nm. A precise thermal gradient was applied, elevating the temperature from 20°C to 95°C with a ramp rate of 0.3°C/min. The generated fluorescence intensity profiles were subsequently processed through sigmoidal regression analysis using the integrated UNcle software suite.

### 4.6 Affinity Determination of Fusion Proteins

The binding kinetics of FD03‐sST2 and its monovalent counterparts (FD03, sST2‐Fc) toward mouse or human ANGPTL3 and murine IL‐33‐His6 were quantified via SPR on a Biacore T200 platform. The respective antigens (Sino Biological, Beijing, China) were covalently tethered to the CM5 sensor chip surface (Fc2 and Fc4 channels). Serial dilutions of the fusion proteins, prepared in HBS‐EP+ running buffer, were delivered across the sensor at 30 µL/min, encompassing an association interval of 120 s and a subsequent 600‐s dissociation period. Surface regeneration between cycles was achieved using 10.0 mM Glycine‐HCl (pH 1.5). The resulting sensorgrams were processed through a 1:1 Langmuir binding algorithm within the Biacore Evaluation framework (version 3.2) to derive the kinetic constants, specifically ka​, kd​, and the overall affinity KD​.

### 4.7 In Vitro LPL Assay

To evaluate the neutralization potency of anti‐ANGPTL3 candidates against ANGPTL3‐mediated LPL suppression, various concentrations of FD03 or FD03‐sST2 were pre‐equilibrated with mANGPTL3 (S17–T206)‐Fc. These mixtures were subsequently introduced to a reaction system containing 50 nM bovine LPL (Yingxin, China) within a specialized assay buffer (pH 8.0, composed of 20 mM Tris, 150 mM NaCl, and 0.2% fatty‐acid‐deficient BSA). The enzymatic reaction was initiated in black 96‐well microplates (Corning, USA) upon the addition of 10 µM fluorogenic Lipase Substrate (Sigma). Kinetic fluorescence profiles (λex = 529 nm; λem = 600 nm) were monitored for 60 min at a constant temperature of 37°C using a BioTek Multi‐Mode detection system.

### 4.8 In Vitro Luciferase Reporter Assay

The inhibitory activity of sST2 on IL‐33 signaling was evaluated using a luciferase reporter assay. sST2‐Fc, FD03‐sST2 and a control antibody at different concentrations were pre‐incubated with hIL‐33‐His10 for 1 h. The mixtures were then added to luciferase reporter cells (2 × 10^4^ cells per well; Suzhou, China) cultured in complete DMEM medium and incubated for 6 h. Luciferase activity was measured in black‐walled 96‐well plates using the Firefly Luciferase Reporter Gene Assay Kit II (RG007S, Beyotime, Shanghai, China).

### 4.9 Animal Study

Five‐week‐old male db/db mice (strain: C57BLKS/JGpt‐Leprem2Cd479/Gpt) and age‐ and sex‐matched male db/m counterparts were sourced from GemPharmatech (Nanjing, China). Environmental maintenance was performed under a specific pathogen‐free (SPF) regime, characterized by a 12‐h circadian light cycle and a steady temperature range (20‐24°C). All animal experimental procedures were reviewed and approved by the Institutional Animal Ethics Committee of the School of Pharmacy, Fudan University (Approval No. 2023‐03‐SY‐JDW‐60), and were conducted in strict accordance with the National Institutes of Health Guide for the Care and Use of Laboratory Animals and the relevant national regulations of China.

Following the induction of DKD via a high‐fat diet (HFD), the db/db cohort was stratified into five distinct treatment arms (n = 6 per group): the DKD vehicle, FD03 monotherapy, sST2‐Fc monotherapy, the FD03‐sST2 fusion protein group, and the FD03 + sST2 combinatorial group. Lean db/m mice (n = 6) on a standard diet served as healthy controls. Therapeutic regimens were administered via intraperitoneal (i.p.) route once weekly for a 6‐week duration. Standardized dosages were calculated based on molar equivalence: 10, 15.9, and 19.4 mg/kg for FD03, sST2‐Fc, and FD03‐sST2, respectively. Body weight was recorded at 7‐day intervals throughout the treatment period. Food intake was monitored weekly by measuring the difference between the amount of food provided and the amount remaining in each cage, and no statistically significant differences in food consumption were observed among treatment groups (Figure S2F).

To monitor disease progression, urinary microalbumin and creatinine (CRE) levels were quantified periodically from 6 weeks of age. Post‐intervention, urine collection was transitioned to a bi‐weekly schedule, while body weights were recorded at 7 day intervals. Upon study completion, mice were humanely euthanized to harvest blood and vital organs (liver and kidney), which were preserved for subsequent molecular and histological evaluations.

### 4.10 Biochemistry Analysis

GLU, TG, TC, LDL‐C, CRE and BUN, as well as urinary albumin and creatinine concentrations, were quantified using commercial assay kits (Nanjing Jiancheng Bioengineering, Nanjing, China).

### 4.11 Cytokine Assay

Serum IL‐1β levels were determined by ELISA (MultiSciences Biotech, Zhejiang, China) following the manufacturer's protocol.

### 4.12 Histopathological Analysis

Liver and kidney tissues were fixed in 4% formaldehyde, paraffin‐embedded, sectioned (5 µm), and stained, or prepared as frozen sections. Three mice per group were analyzed. H&E staining assessed renal injury. PAS staining evaluated glomerular mesangial expansion (mesangial index). Masson's trichrome staining assessed collagen deposition. Oil Red O staining on frozen sections evaluated neutral lipid accumulation. Images were captured with SlideView VS200 (Olympus) and quantified using ImageJ (NIH, Bethesda, USA).

### 4.13 Immunohistochemical Analysis

Fibronectin, α‐SMA, and NLRP3 expression were detected using rabbit monoclonal antibodies: anti‐fibronectin (ab268020, clone EPR20994, 1:200 dilution), anti‐α‐SMA (ab108424, clone EPR5368, 1:500 dilution), and anti‐NLRP3 (ab263899, clone EPR23094‐120, 1:200 dilution), all sourced from Abcam (Cambridge, UK). Nuclei were counterstained with DAPI (ab285390, Abcam, 1:1000 dilution). Positively stained areas were quantified from SlideView VS200 images using ImageJ.

### 4.14 Confocal Immunofluorescence Microscopy

To visualize the renal expression of IL‐33 and the spatial localization of GATA3‐positive ILC2s, kidney sections underwent immunofluorescence labeling. Primary antibodies targeting IL‐33 (28035, rabbit polyclonal, 1:200 dilution) and GATA3 (66400, rabbit polyclonal, 1:200 dilution), both sourced from Proteintech (Wuhan, China), were applied to the tissues. Sections were subsequently incubated with Alexa Fluor 488‐conjugated goat anti‐rabbit IgG secondary antibody (for IL‐33, green channel) and Alexa Fluor 555‐conjugated goat anti‐rabbit IgG secondary antibody (for GATA3, red channel) (1:500 dilution; Thermo Fisher Scientific, USA). Nuclei were identified via DAPI counterstaining. High‐resolution fluorescent images were acquired using an LSM 980 confocal laser scanning platform (Carl Zeiss Inc., Oberkochen, Germany).

### 4.15 Transcriptomic Profiling and Bioinformatic Pipelines

Following the extraction of total RNA from renal specimens, the quantity and molecular integrity were rigorously validated. High‐quality libraries were subsequently synthesized and subjected to high‐throughput sequencing via the Illumina NovaSeq 6000 system. The raw reads were mapped against the murine reference sequence (Ensembl GRCm38/mm10). To discern transcriptional variations, differentially expressed genes (DEGs) were filtered through the DESeq2 algorithm, applying a significance criterion of *P* < 0.05. Functional annotation, including Gene Ontology (GO) and KEGG pathway enrichment, was executed utilizing the Goatools and Python Scipy frameworks to elucidate the biological implications of the identified DEGs.

### 4.16 Untargeted Metabolomic and Lipidomic Profiling

Renal tissues were prepared for downstream analysis adhering to established protocols. Chromatographic separation of diverse metabolites and lipid species was achieved using a series of specialized columns (T3, BEH, and C18; Waters and Phenomenex) integrated with an ExionLC 2.0 ultra‐high‐performance liquid chromatography (UHPLC) system. Mass spectrometric detection was subsequently executed on an AB SCIEX X500B platform. The experimental cohort, comprising 48 individual data acquisitions (including 36 biological replicates, 6 quality control pools, and 6 blank controls), was processed by converting raw files into mzXML/mgf formats via the ProteoWizard toolkit. Peak intensities were derived using a specialized data‐mining software (version 2023SR0256527). Chemical identification of metabolites was primarily facilitated through the MetDNA2 web‐server, supplemented by Lipid4DAnalyzer for lipidomic annotation. Final multivariate statistical evaluations were conducted using the MetaboAnalyst 5.0 computational framework.

### 4.17 Western Blot

Following the isolation of total protein from renal tissues, concentrations were determined utilizing a BCA colorimetric suite (Yeasen, China). Aliquots containing 20 µg of protein were resolved via SDS–PAGE and subsequently electrotransferred onto membranes for immunoprobing. The following primary rabbit or mouse monoclonal antibodies were employed: NF‐κB p65 (#8242), p‐NF‐κB p65 (#3033), IκBα (#4812), and p‐IκBα (#2859) from Cell Signaling Technology, alongside caspase‐1 (ab179515, Abcam) and GAPDH (#2118S, CST) as a loading control. Species‐specific secondary antibodies (Proteintech, China) were applied for signal detection. Protein signatures were visualized through enhanced chemiluminescence (ECL; Meilunbio, China) and quantified by densitometric scanning using the ImageJ software package (version 1.8.0, NIH).

### 4.18 Quantitative Real‐Time PCR (RT‐qPCR) Analysis

Following RNA concentration assessment via a NanoDrop spectrophotometer, the isolated RNA was converted into complementary DNA (cDNA) utilizing specialized reverse transcription modules (R333 and Q711; Vazyme Biotech, Nanjing, China). Quantitative amplification was subsequently performed on a QuantStudio 3 real‐time PCR platform (Thermo Fisher Scientific, USA). The specific oligonucleotide primer sequences employed in this study are detailed in Table 1.

**TABLE 1:** Primer pairs used for vector construction.

Primer	Sequence forward	Sequence reverse
GAPDH	GTCCTCAGTGTAGCCCAAGATG	CAATGTGTCCGTCGTGGATCT
IL‐1β	ACCTGTGTCTTTCCCGTGG	TCATCTCGGAGCCTGTAGTG
NLRP3	CCCTTGGAGACACAGGACTC	GAGGCTGCAGTTGTCTAATTCC
TGF‐β	GTGTGGAGCAACATGTGGAACTCT	CGCTGAATCGAAAGCCCTGTA
Fibronectin	ATACCGTTGTCCCAGAGGTG	GGAAGAGTTTAGCGGGGTCC
α‐SMA	GCTGGACTCTGGAGATGGTG	CAATCTCACGCTCGGCAGTA
Collage I	GAGGCCTCCCAGAACATCAC	AAGTTCCGGTGTGACTCGTG

### 4.19 Isolation of ILC2s From Murine Kidney

To isolate ILC2s, kidneys harvested from 8‐week‐old female naïve C57BL/6 mice (B&K Universal Group, China) underwent transcardial perfusion with PBS followed by mechanical fragmentation. The resultant tissue segments were subjected to enzymatic digestion at 37°C for 60 min in RPMI 1640 medium supplemented with 10% heat‐inactivated FBS, Collagenase I, and Deoxyribonuclease I (Yeasen, China). Single‐cell suspensions were obtained by passing the digest through a 70‐µm nylon strainer, with subsequent erythrocyte depletion using a specialized lysis reagent (Servicebio, China). Following a wash step, the clarified cell pellet was reconstituted in complete medium. The target ILC2 population was subsequently isolated via negative selection using the EasySep Mouse ILC2 Enrichment Kit (Stem Cell Technologies, Canada) in strict accordance with the stipulated technical guidelines.

### 4.20 In Vitro Bioactivity of IL‐33 in Murine Kidney ILC2s

Freshly isolated murine renal ILC2s were distributed into 96‐well flat‐bottomed microplates at a concentration of 5 × 10^4^ cells/well. The culture environment consisted of RPMI 1640 medium enriched with 10% FBS, 1% antibiotic‐antimycotic solution, and 1× nonessential amino acids (Yeasen, China). To assess the inhibitory effects, mIL‐33‐His6 was pre‐equilibrated with FD03, sST2‐Fc, or the FD03‐sST2 fusion protein for 60 min prior to cell administration. Following a 12 h stimulation period, the cellular fractions were harvested to quantify transcriptional variations via RT‐qPCR analysis.

### 4.21 Statistical Analysis

Quantitative results are expressed as mean ± SEM. To determine statistical disparities across multiple cohorts, a one‐way ANOVA was implemented, with post–hoc evaluations conducted via Tukey's range test. Differences were deemed to possess statistical significance when the associated *p*‐values fell below 0.05. The magnitude of significance is denoted in the figures as follows: ^*^ *p* < 0.05, ^**^ *p* < 0.01, ^***^ *p* < 0.001, ^****^ *p* < 0.0001.

## Author Contributions

Z.J.L. performed the experiments and wrote the original draft. Z.C. and Z.L.C. and R.R.Z. participated in study design and manuscript revision. X.C., X.Z.H., and S.W.X. contributed significantly to protein engineering and in vivo experimental design. Z.H.D. provided technical assistance with metabolomic profiling. Y.S. and L.D. contributed to experimental design and secured resources, including equipment and funding. D.W.J. conceptualized the study, supervised the research, revised the manuscript, and acquired funding and resources.

## Conflicts of Interest

The authors declare no conflict of interest.

## Supporting information




**Supporting File**: advs75756‐sup‐0001‐SuppMat.pdf.

## Data Availability

The data that support the findings of this study are available in the supplementary material of this article.
