# Supplementary material for: Targeting ANGPTL3 and IL‐33/ST2 Ameliorates Diabetic Kidney Disease by Reducing Lipotoxicity, Alleviating Inflammation and Inhibiting Fibrosis
Source: Adv Sci (Weinh). 2026 May 19;13(45):e75756. doi: 10.1002/advs.75756 (PMC13336007; doi:10.1002/advs.75756)
Supplement: Supplementary file 1 [file ADVS-13-e75756-s001.pdf]

SUPPLEMENTARY INFORMATION

Targeting ANGPTL3 and IL-33/ST2 Ameliorates Diabetic Kidney Disease by Reducing Lipotoxicity, Alleviating Inflammation and Inhibiting Fibrosis

Zhuojin Li et al.

Ling Du. Email: mduling1981@163.com.

Dianwen Ju. Email: dianwenju@fudan.edu.cn

Supplementary figure 1

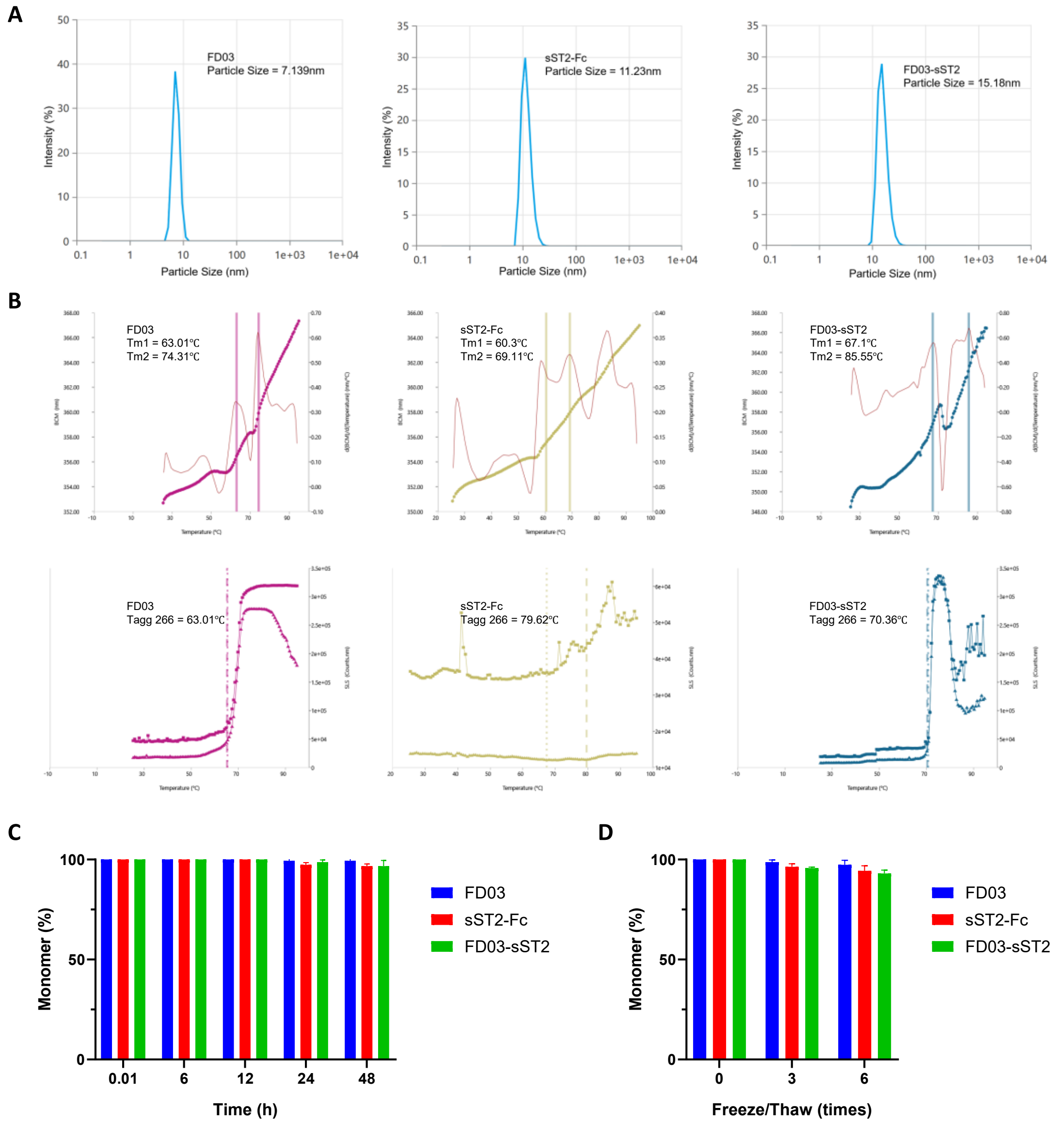

Supplementary figure 1. Characterization and stability of FD03-sST2.

(A) Representative images of size distribution of FD03, sST2-Fc and FD03-sST2. (B) Thermal stability analysis of FD03, sST2-Fc and FD03-sST2. (C) The serum stability of FD03, sST2-Fc and FD03-sST2 by SEC-HPLC. (D) The freeze-thaw stability analysis of FD03, sST2-Fc and FD03-sST2 by SEC-HPLC.

Supplementary figure 2

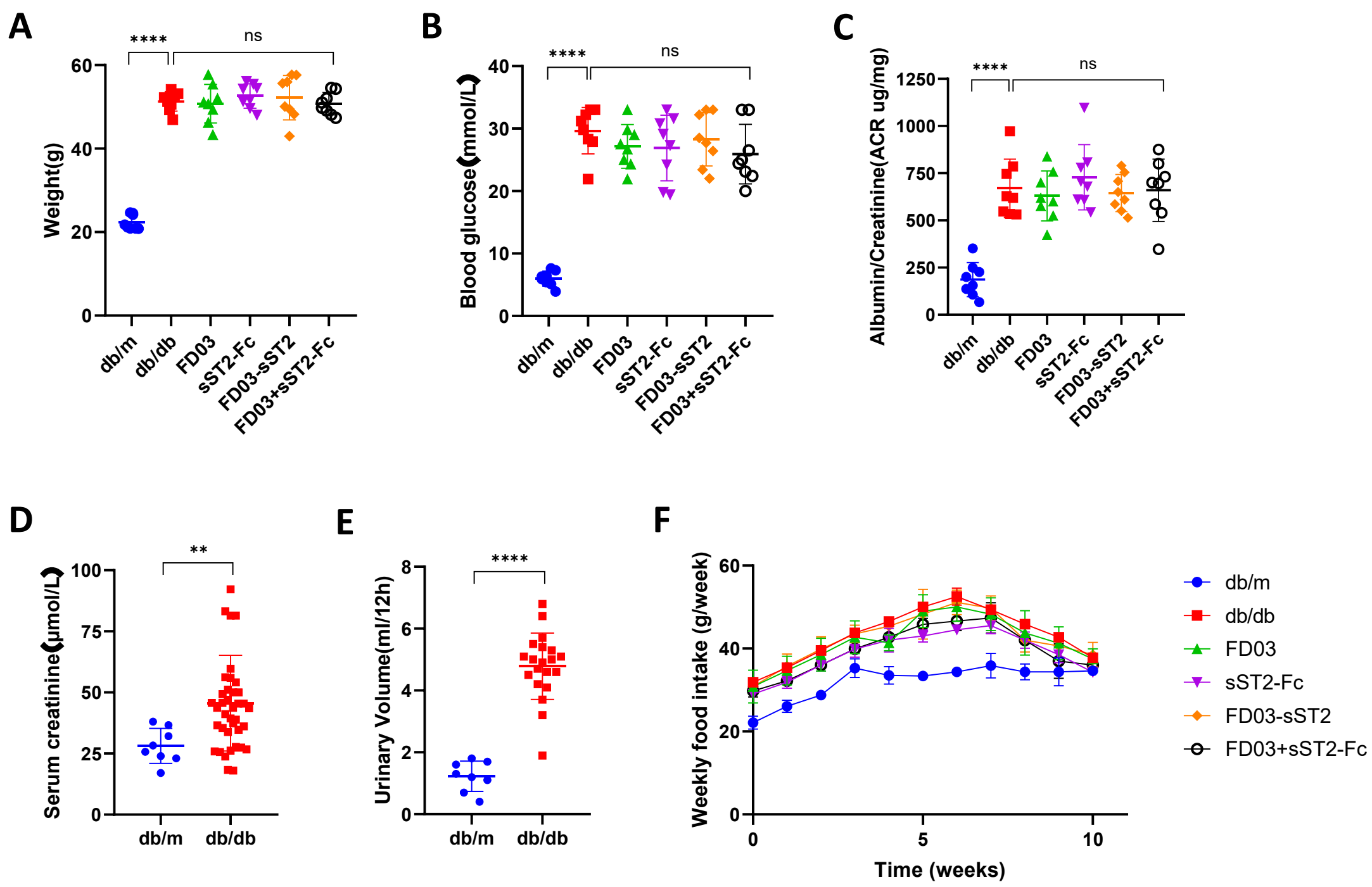

Supplementary figure 2. Establishment of the DKD mouse model.

(A-E) Following 4 weeks of high-fat diet feeding, db/db mice exhibited hallmark features of DKD, including significantly elevated weight, and GLU (A, B), as well as increases in ACR, serum CRE, and 12-hour urine volume (C-E), confirming successful model establishment (n = 8). (F) Statistical analysis of weekly food intake of mice in each treatment group for 0-10 weeks. The data in (A-E) are presented as mean ± SD. ****$P < 0.0001$, ***$P < 0.001$, **$P < 0.01$, *$P < 0.05$; ns, not significant. Statistical significance was assessed by one-way ANOVA with Tukey's multiple-comparisons test.

Supplementary figure 3.

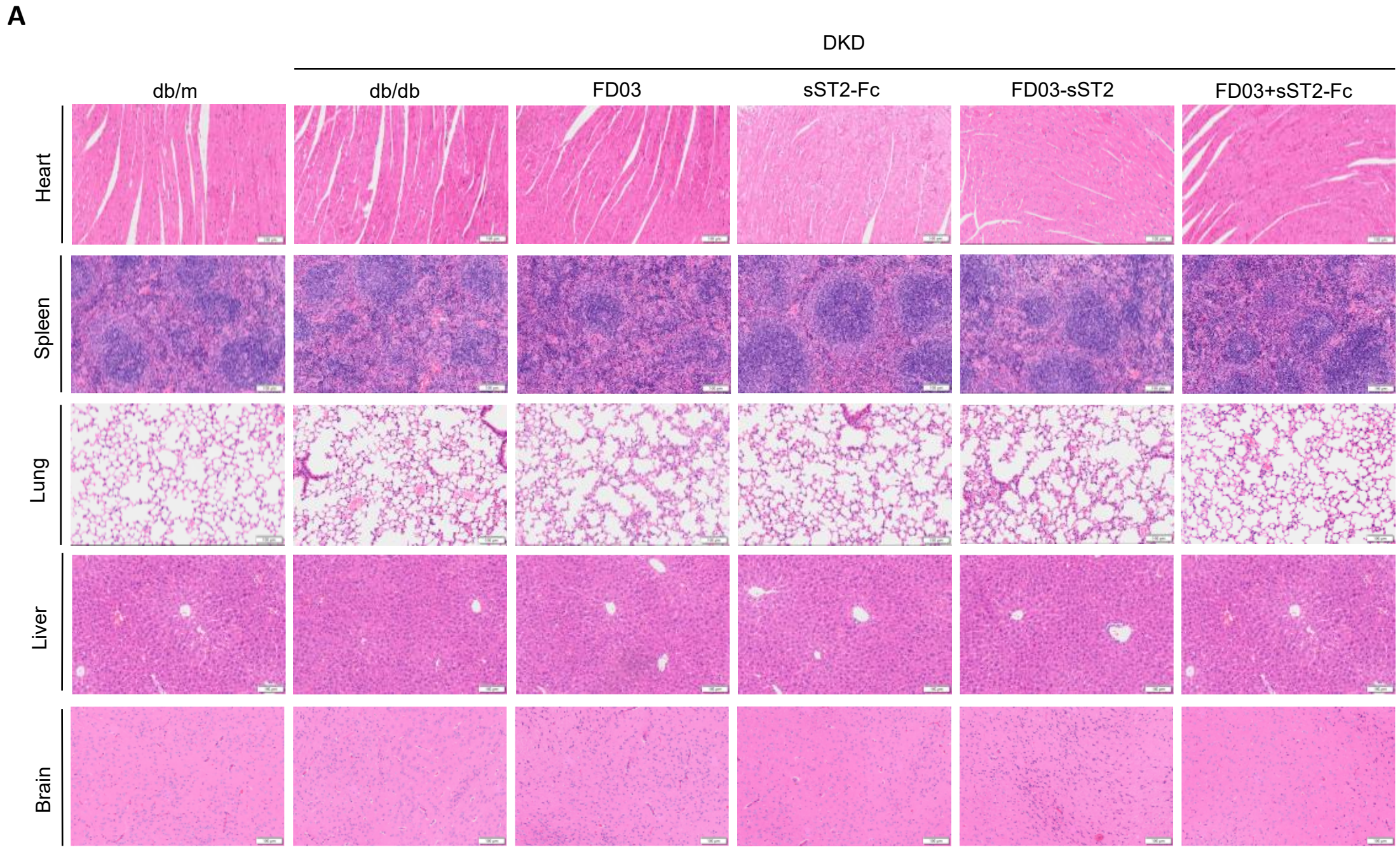

Supplementary figure 3. Evaluation of the safety profile of FD03-sST2 in various tissues.

(A) Representative H&E staining images of heart, spleen, lung, liver and brain tissues with FD03, sST2-Fc, FD03-sST2 and combination administration (scale bar = 100μm).

Supplementary figure 4.

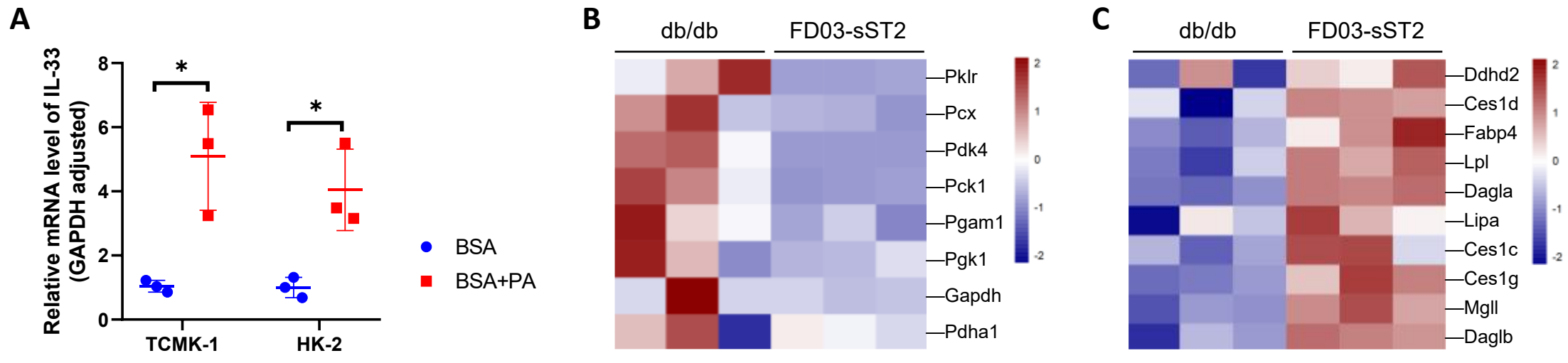

Supplementary Figure 4. Cellular mechanisms and genetic validation of FD03-sST2-mediated improvement of renal glucose and lipid metabolism in DKD.

(A) IL-33 expression in TCMK-1 and HK-2 cells stimulated with 0.4 mM palmitic acid (PA) for 24 h (n = 3). (B, C) Heatmaps of genes involved in gluconeogenesis (B) and triglyceride metabolism (C) in kidneys from db/db mice versus FD03-sST2-treated db/db mice. The data in (A) are presented as mean ± SD. *****P* < 0.0001, ****P* < 0.001, ***P* < 0.01, **P* < 0.05; ns, not significant. Statistical significance was assessed by one-way ANOVA with Tukey's multiple-comparisons test.
